# Supplementary material for: The Integrated Bioinformatic Approach Reveals the Prognostic Significance of LRP1 Expression in Ovarian Cancer
Source: Int J Mol Sci. 2024 Jul 22;25(14):7996. doi: 10.3390/ijms25147996 (PMC11276689; doi:10.3390/ijms25147996)
Supplement: Supplementary file 1 [file ijms-25-07996-s001.zip › ijms-3078802-supplementary.pdf]

The integrated bioinformatic approach reveals the Prognostic significance of LRP1 Expression in Ovarian Cancer

Tesfaye Wolde[1†], Vipul Bhardwaj[2†], Md. Reyad-ul-Ferdous[1], Peiwu Qin[1,2*] and Vijay Pandey[1,2*]

[1]Institute of Biopharmaceutical and Health Engineering, Tsinghua Shenzhen International Graduate School, Tsinghua University, Shenzhen 518055, China; tesfalem2002@gmail.com (T.W.); bhardwajvipul27@yahoo.in (V.B.); rockyreyad@sz.tsinghua.edu.cn (M.R.F.)

[2]Tsinghua Berkeley Shenzhen Institute, Tsinghua Shenzhen International Graduate School, Tsinghua University, Shenzhen 518055, China.

[†]*Authors contributed equally*

**Corresponding to:* Vijay Pandey, Tsinghua Shenzhen International Graduate School, Nanshan, Shenzhen, 518055, China, Email: vijay.pandey@sz.tsinghua.edu.cn and Peiwu Qin, Institute of Biopharmaceutical and Health Engineering, Tsinghua Shenzhen International Graduate School, Tsinghua University, Shenzhen 518055, PR China, Email: pwqin@sz.tsinghua.edu.cn.

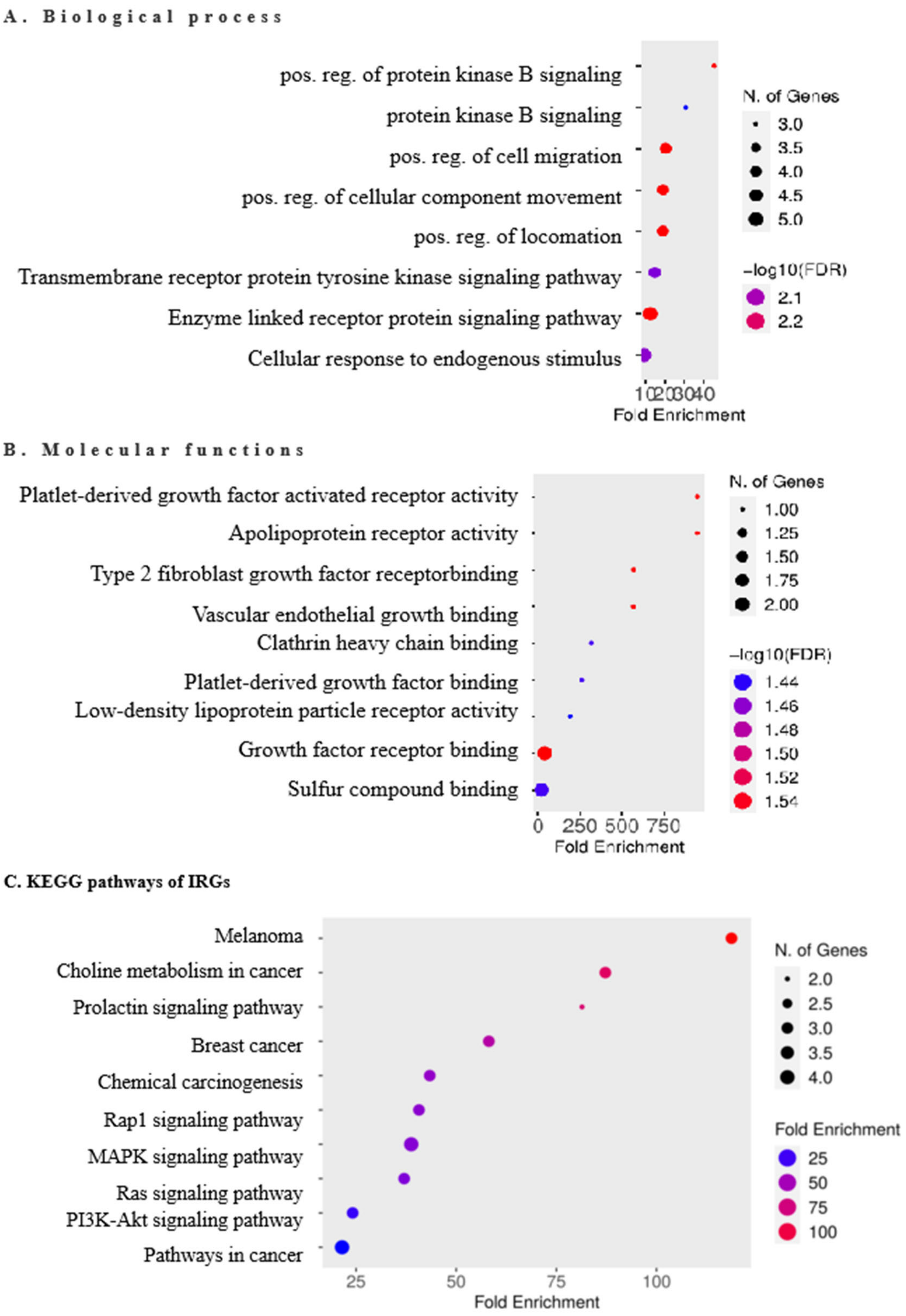

Supplementary Figure S1. Bar chart of Gene Ontology (GO) enriched in Biological Process:

A. **Bar chart of Gene Ontology (GO) enriched in Biological Process:** The bar chart represents the enrichment of immune related genes in various biological processes (BP). The analysis was conducted using ShinyGO 0.80, revealing significant biological processes in which the candidate IRGS are involved. The x-axis shows the GO terms related to biological processes, while the y-axis

indicates the number of genes associated with each term. The most enriched biological processes include those related to cell proliferation, apoptosis, signal transduction, and immune response.

B. **Bar chart of Gene Ontology (GO) enriched in Molecular Functions:** The bar chart illustrates the enrichment of genes in different molecular functions (MF). The x-axis lists the GO terms associated with molecular functions, and the y-axis represents the number of genes involved. Key molecular functions identified include binding activities, such as protein binding, ATP binding, and receptor binding, as well as catalytic activities like enzyme activity and DNA-binding transcription factor activity.

C. **Bar chart of KEGG Pathway Enrichment Analysis:** The bar chart presents the KEGG pathway enrichment analysis, indicating the pathways significantly associated with ovarian cancer. The x-axis lists the KEGG pathways, and the y-axis indicates the number of genes enriched in each pathway. Key pathways identified include signal transduction pathways, immune system pathways, cancer-related pathways, and metabolic pathways. This analysis provides insights into the molecular mechanisms and pathways through which the candidate genes may contribute to ovarian cancer pathogenesis and progression.

A. Relation between clinicopathological features stromal score.

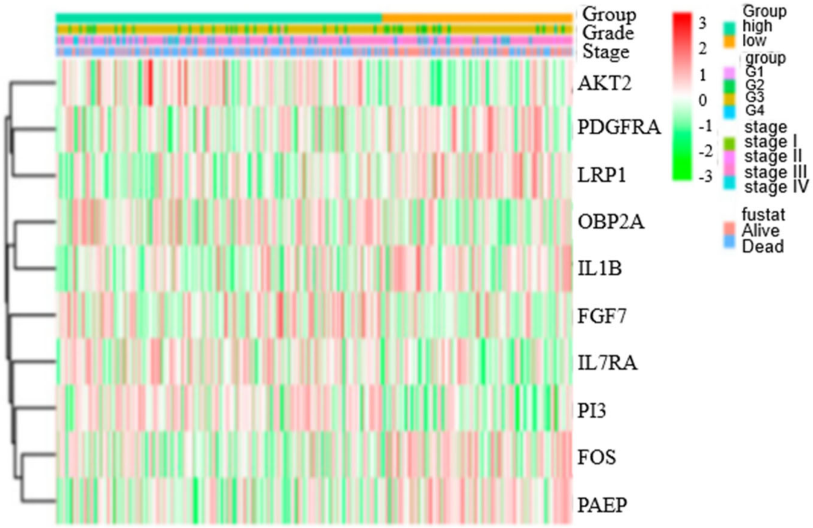

Supplementary Figure S2. Clinicopathological Features and Stromal Score

A. **Relation Between Clinicopathological Features and Stromal Score:** This section illustrates the relationship between various clinicopathological features and the stromal score in ovarian cancer. The stromal score, which reflects the presence of stromal cells in the tumour microenvironment, is analyzed in relation to different clinical and pathological characteristics of the patients, highlighting its potential impact on disease progression and patient prognosis. These analyses provide valuable insights into the complex interactions between IRGs, TMB, MSI, and the tumour microenvironment in ovarian cancer, emphasizing the significance of these genes in cancer development and potential therapeutic strategies.

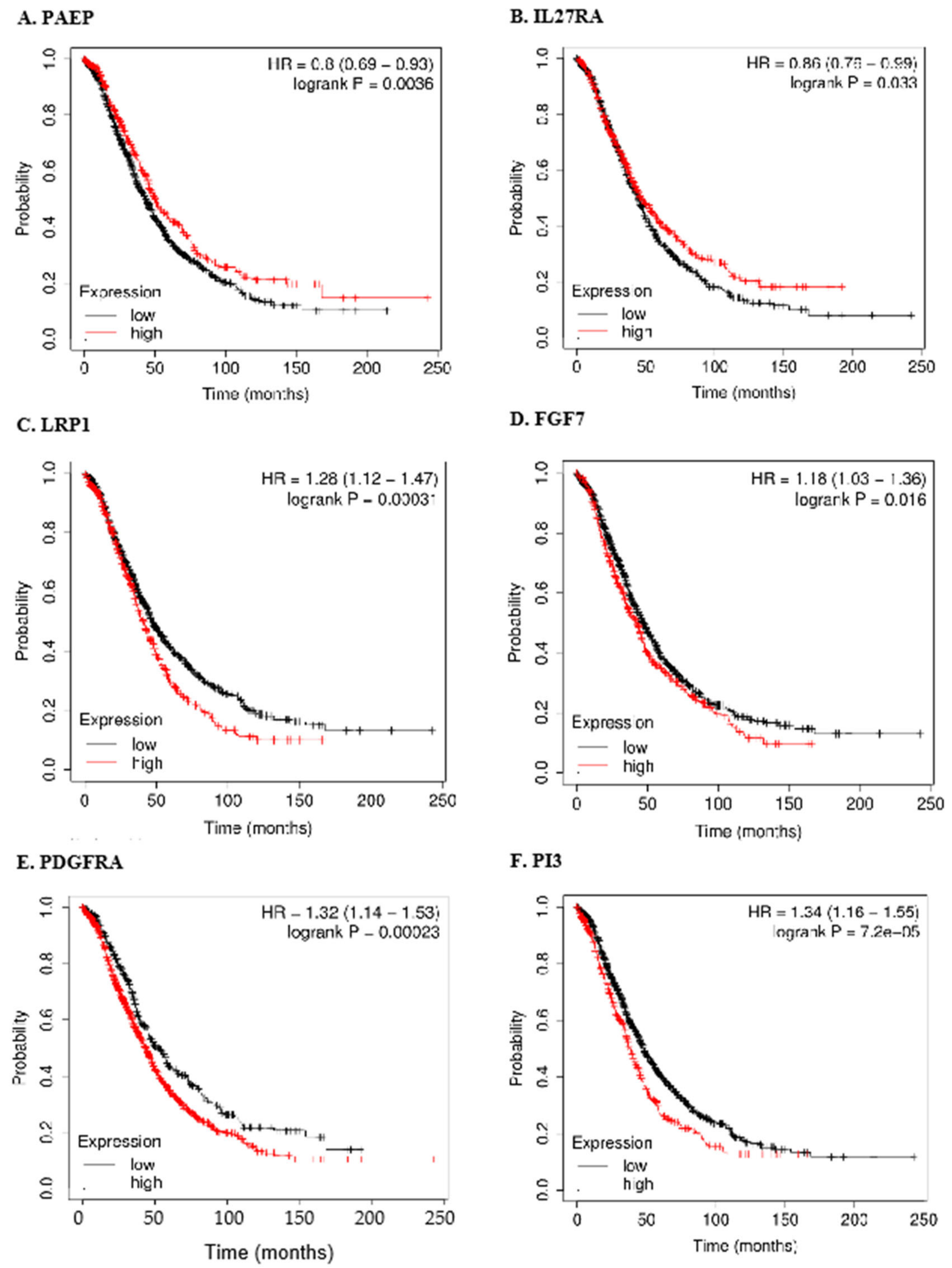

Supplementary Figure S3. The Prognostic Effect of IRGs Expression: presents Kaplan-Meier survival curve analyses of overall survival (OS) stratified by high-risk (red curve) and low-risk (black curve) groups, based on the expression levels of various immune-related genes (IRGs). The analysis was conducted using data from www.kmplot.com.

A. **PAEP:** Kaplan-Meier survival curves comparing high and low expression levels of PAEP. The red curve represents patients with high PAEP expression, while the black curve represents those with low PAEP expression.

B. **IL27RA:** Kaplan-Meier survival curves for IL27RA. The red curve indicates high expression, and the black curve indicates low expression, showing the prognostic impact on OS.

C. **LRP1:** Kaplan-Meier survival curves for LRP1. The red curve denotes high LRP1 expression, and the black curve denotes low LRP1 expression, illustrating the difference in OS between the two groups.

D. **FGF7:** Kaplan-Meier survival curves for FGF7 expression levels. High expression is represented by the red curve and low expression by the black curve, highlighting its prognostic significance.

E. **PDGFRA:** Kaplan-Meier survival curves for PDGFRA. High expression is indicated by the red curve, and low expression by the black curve, showing the correlation with OS.

F. **PI3:** Kaplan-Meier survival curves for PI3 expression. The red curve represents high PI3 expression, and the black curve represents low PI3 expression, demonstrating its impact on OS. These Kaplan-Meier plots emphasize the prognostic significance of PAEP, IL27RA, LRP1, FGF7, PDGFRA, and PI3 expression levels in determining the overall survival of patients, with high-risk groups consistently showing poorer outcomes.

A. AKT2

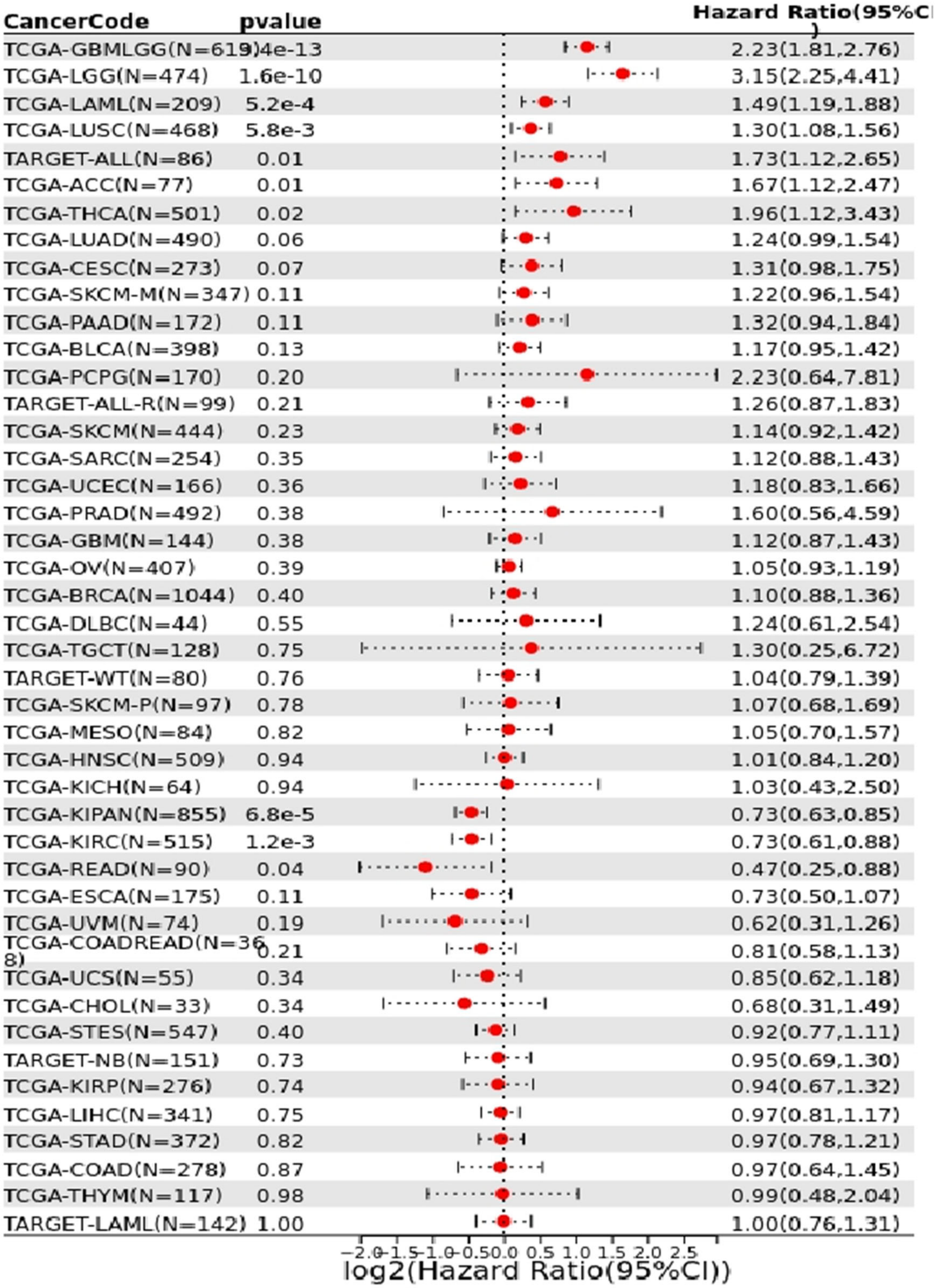
CancerCode
pvalue
Hazard Ratio(95%CI)
TCGA-GBMLGG(N=619) 9.4e-13 2.23(1.81,2.76)
TCGA-LGG(N=474) 1.6e-10 3.15(2.25,4.41)
TCGA-LAML(N=209) 5.2e-4 1.49(1.19,1.88)
TCGA-LUSC(N=468) 5.8e-3 1.30(1.08,1.56)
TARGET-ALL(N=86) 0.01 1.73(1.12,2.65)
TCGA-ACC(N=77) 0.01 1.67(1.12,2.47)
TCGA-THCA(N=501) 0.02 1.96(1.12,3.43)
TCGA-LUAD(N=490) 0.06 1.24(0.99,1.54)
TCGA-CESC(N=273) 0.07 1.31(0.98,1.75)
TCGA-SKCM-M(N=347) 0.11 1.22(0.96,1.54)
TCGA-PAAD(N=172) 0.11 1.32(0.94,1.84)
TCGA-BLCA(N=398) 0.13 1.17(0.95,1.42)
TCGA-PCPG(N=170) 0.20 2.23(0.64,7.81)
TARGET-ALL-R(N=99) 0.21 1.26(0.87,1.83)
TCGA-SKCM(N=444) 0.23 1.14(0.92,1.42)
TCGA-SARC(N=254) 0.35 1.12(0.88,1.43)
TCGA-UCEC(N=166) 0.36 1.18(0.83,1.66)
TCGA-PRAD(N=492) 0.38 1.60(0.56,4.59)
TCGA-GBM(N=144) 0.38 1.12(0.87,1.43)
TCGA-OV(N=407) 0.39 1.05(0.93,1.19)
TCGA-BRCA(N=1044) 0.40 1.10(0.88,1.36)
TCGA-DLBC(N=44) 0.55 1.24(0.61,2.54)
TCGA-TGCT(N=128) 0.75 1.30(0.25,6.72)
TARGET-WT(N=80) 0.76 1.04(0.79,1.39)
TCGA-SKCM-P(N=97) 0.78 1.07(0.68,1.69)
TCGA-MESO(N=84) 0.82 1.05(0.70,1.57)
TCGA-HNSC(N=509) 0.94 1.01(0.84,1.20)
TCGA-KICH(N=64) 0.94 1.03(0.43,2.50)
TCGA-KIPAN(N=855) 6.8e-5 0.73(0.63,0.85)
TCGA-KIRC(N=515) 1.2e-3 0.73(0.61,0.88)
TCGA-READ(N=90) 0.04 0.47(0.25,0.88)
TCGA-ESCA(N=175) 0.11 0.73(0.50,1.07)
TCGA-UVM(N=74) 0.19 0.62(0.31,1.26)
TCGA-COADREAD(N=368) 0.21 0.81(0.58,1.13)
TCGA-UCS(N=55) 0.34 0.85(0.62,1.18)
TCGA-CHOL(N=33) 0.34 0.68(0.31,1.49)
TCGA-STES(N=547) 0.40 0.92(0.77,1.11)
TARGET-NB(N=151) 0.73 0.95(0.69,1.30)
TCGA-KIRP(N=276) 0.74 0.94(0.67,1.32)
TCGA-LIHC(N=341) 0.75 0.97(0.81,1.17)
TCGA-STAD(N=372) 0.82 0.97(0.78,1.21)
TCGA-COAD(N=278) 0.87 0.97(0.64,1.45)
TCGA-THYM(N=117) 0.98 0.99(0.48,2.04)
TARGET-LAML(N=142) 1.00 1.00(0.76,1.31)
−2.0 −1.5 −1.0 −0.5 0.0 0.5 1.0 1.5 2.0 2.5
log2(Hazard Ratio(95%CI))

B. PI3

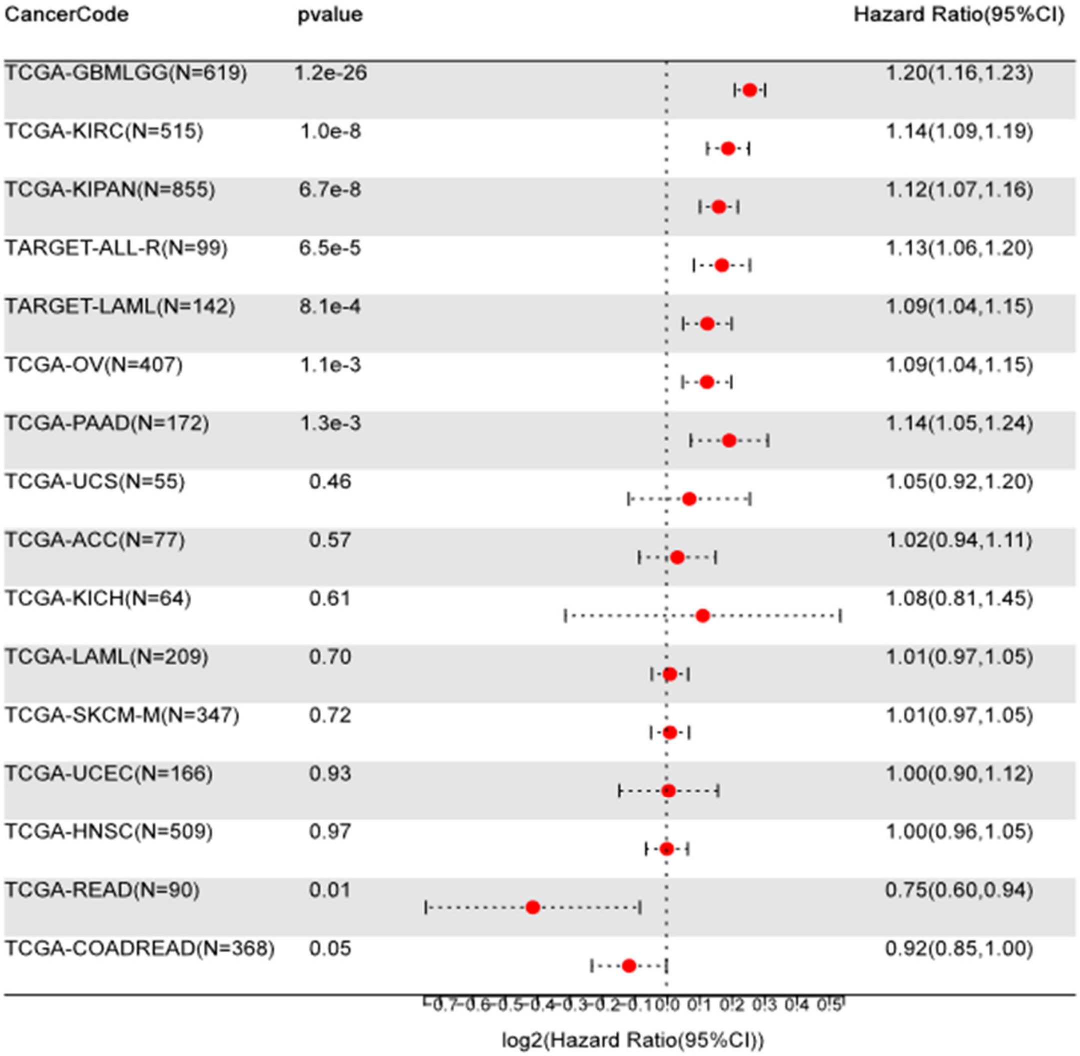

CancerCode
pvalue
Hazard Ratio(95%CI)
TCGA-GBMLGG(N=619) 1.2e-26 1.20(1.16,1.23)
TCGA-KIRC(N=515) 1.0e-8 1.14(1.09,1.19)
TCGA-KIPAN(N=855) 6.7e-8 1.12(1.07,1.16)
TARGET-ALL-R(N=99) 6.5e-5 1.13(1.06,1.20)
TARGET-LAML(N=142) 8.1e-4 1.09(1.04,1.15)
TCGA-OV(N=407) 1.1e-3 1.09(1.04,1.15)
TCGA-PAAD(N=172) 1.3e-3 1.14(1.05,1.24)
TCGA-UCS(N=55) 0.46 1.05(0.92,1.20)
TCGA-ACC(N=77) 0.57 1.02(0.94,1.11)
TCGA-KICH(N=64) 0.61 1.08(0.81,1.45)
TCGA-LAML(N=209) 0.70 1.01(0.97,1.05)
TCGA-SKCM-M(N=347) 0.72 1.01(0.97,1.05)
TCGA-UCEC(N=166) 0.93 1.00(0.90,1.12)
TCGA-HNSC(N=509) 0.97 1.00(0.96,1.05)
TCGA-READ(N=90) 0.01 0.75(0.60,0.94)
TCGA-COADREAD(N=368) 0.05 0.92(0.85,1.00)
log2(Hazard Ratio(95%CI))

C. PAEP

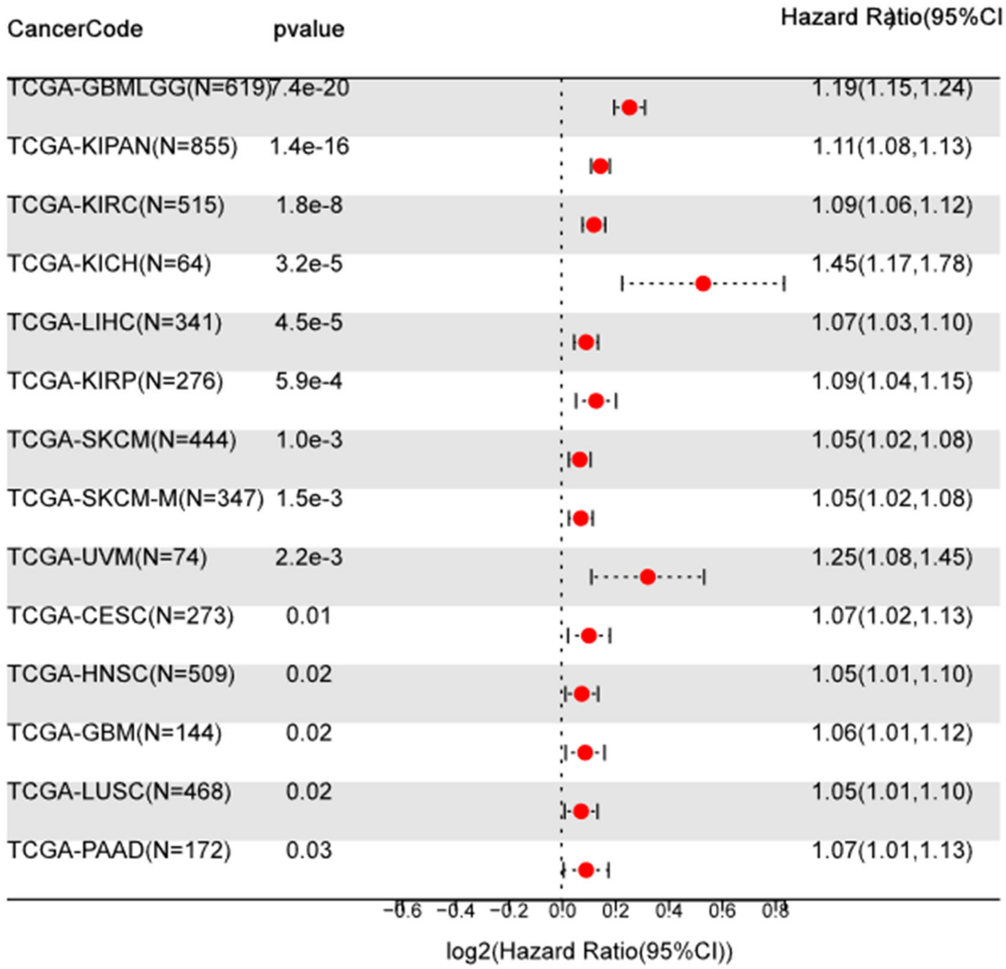

CancerCode
pvalue
Hazard Ratio(95%CI)
TCGA-GBMLGG(N=619)
7.4e-20
1.19(1.15,1.24)
TCGA-KIPAN(N=855)
1.4e-16
1.11(1.08,1.13)
TCGA-KIRC(N=515)
1.8e-8
1.09(1.06,1.12)
TCGA-KICH(N=64)
3.2e-5
1.45(1.17,1.78)
TCGA-LIHC(N=341)
4.5e-5
1.07(1.03,1.10)
TCGA-KIRP(N=276)
5.9e-4
1.09(1.04,1.15)
TCGA-SKCM(N=444)
1.0e-3
1.05(1.02,1.08)
TCGA-SKCM-M(N=347)
1.5e-3
1.05(1.02,1.08)
TCGA-UVM(N=74)
2.2e-3
1.25(1.08,1.45)
TCGA-CESC(N=273)
0.01
1.07(1.02,1.13)
TCGA-HNSC(N=509)
0.02
1.05(1.01,1.10)
TCGA-GBM(N=144)
0.02
1.06(1.01,1.12)
TCGA-LUSC(N=468)
0.02
1.05(1.01,1.10)
TCGA-PAAD(N=172)
0.03
1.07(1.01,1.13)
−0.6
−0.4
−0.2
0.0
0.2
0.4
0.6
0.8
log2(Hazard Ratio(95%CI))

D. IL27RA

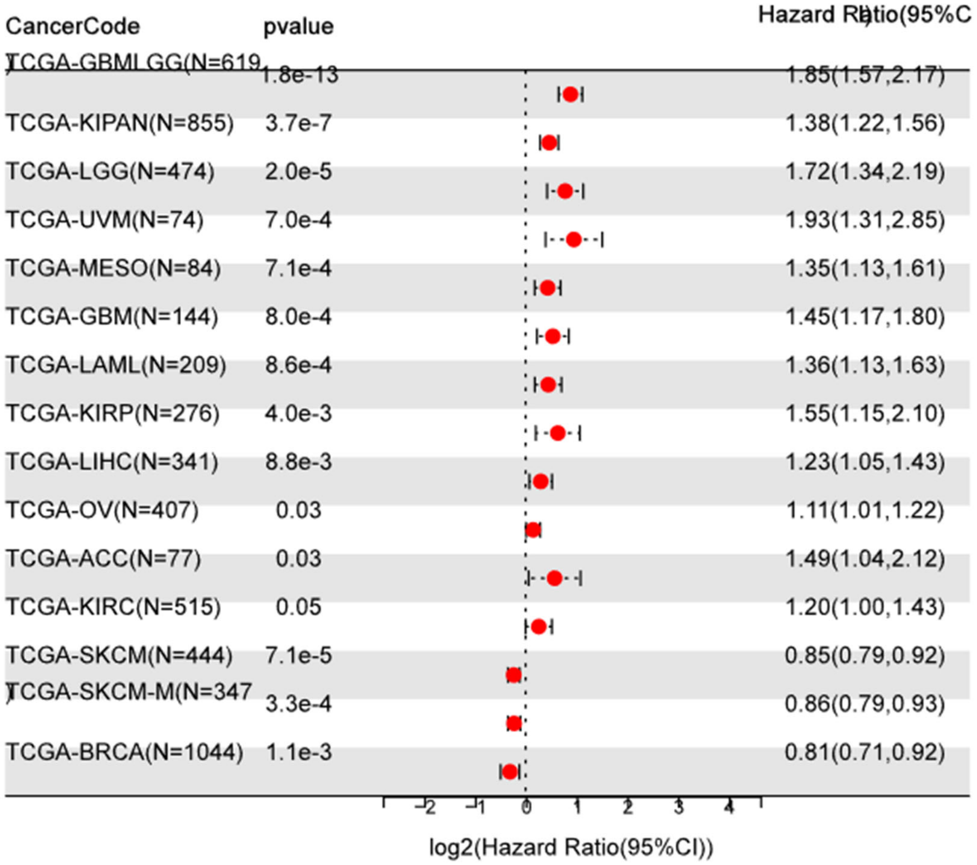

E. OBP2A

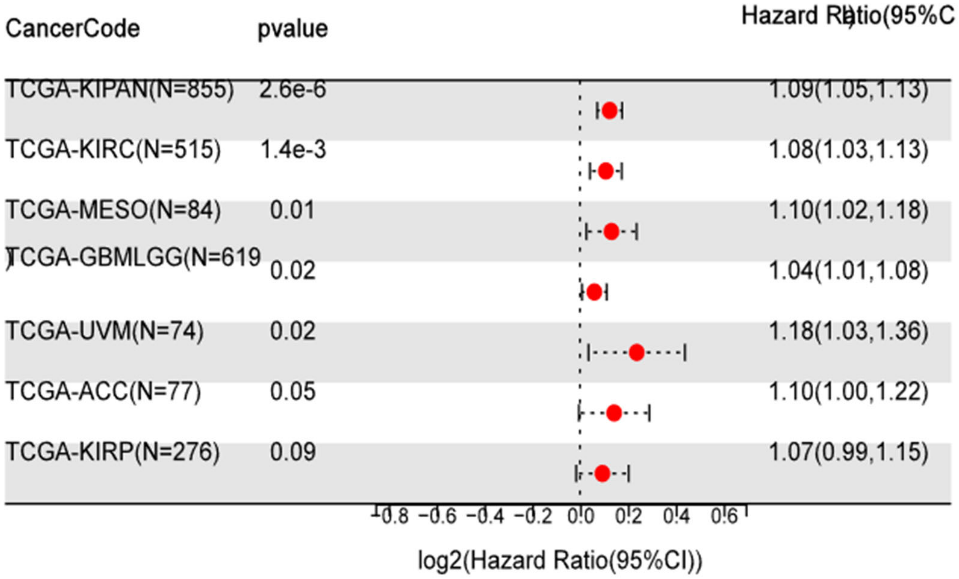

F. PDGFRA

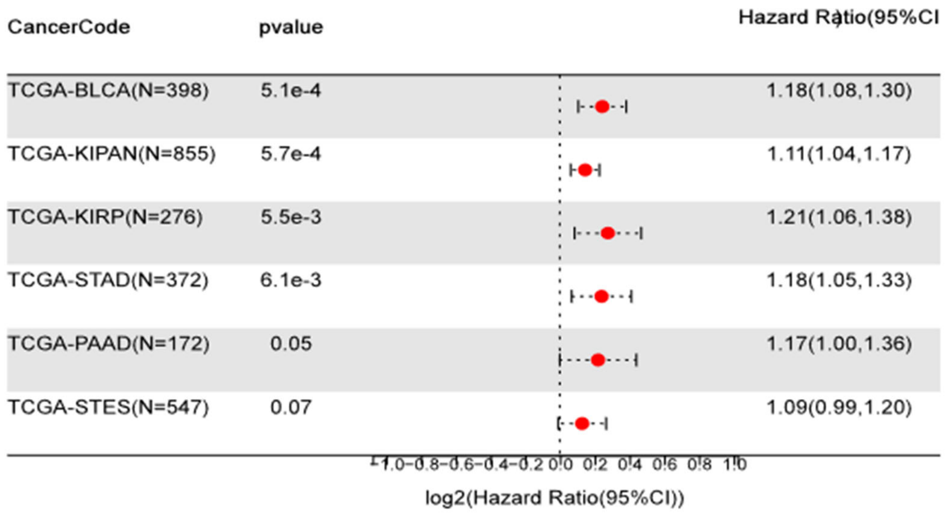

G. FOS

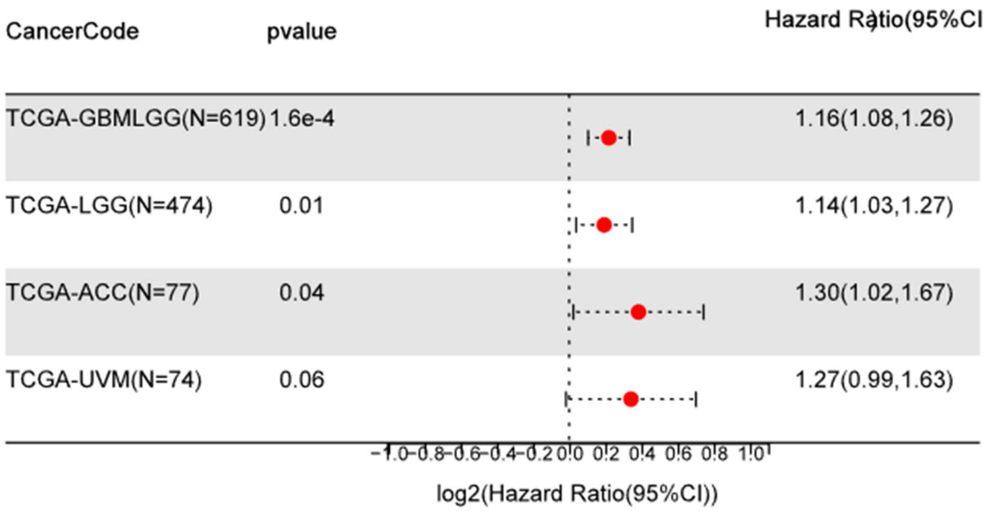

H. LRP1

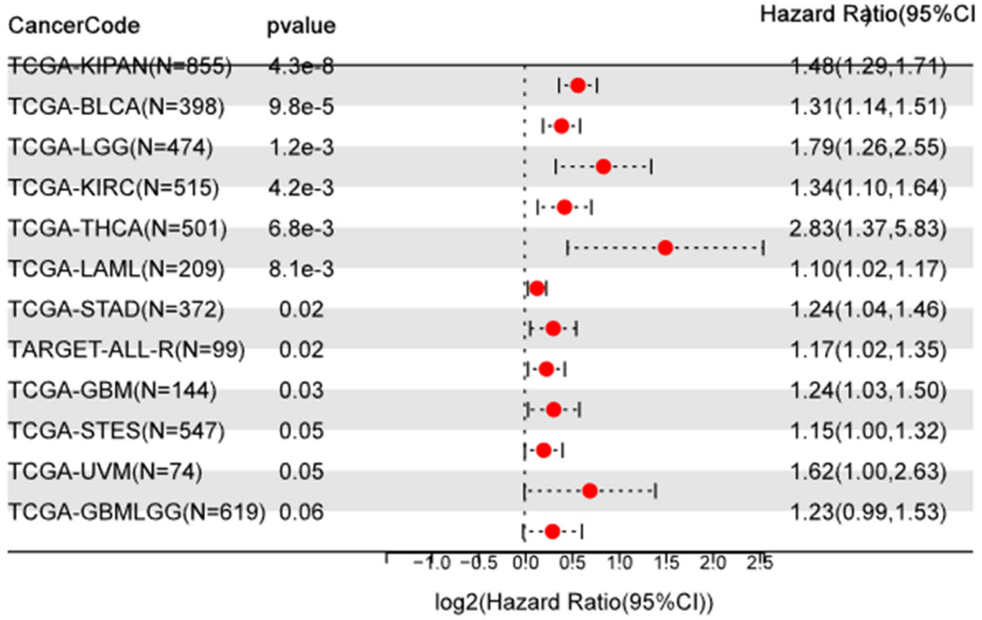

I. FGF7

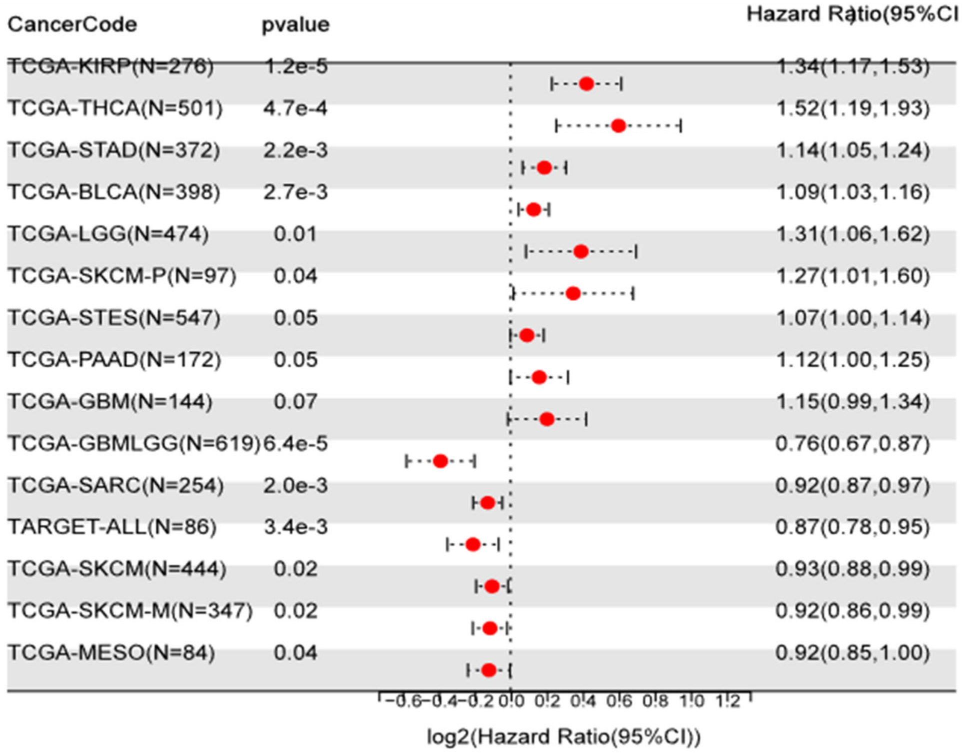

Supplementary Figure S4. Forest Plots of the Association Between IRGs Expression and Overall Survival (OS) in 33 Cancer Types.

A. **AKT2 Expression:** A forest plot showing the association between AKT2 expression and OS. High expression of AKT2 is linked to poor prognosis in adrenocortical carcinoma (ACC), ovarian cancer

(OC), liver hepatocellular carcinoma (LIHC), kidney renal papillary cell carcinoma (KIRP), lower-grade glioma (LGG), acute myeloid leukemia (LAML), glioblastoma multiforme (GBM), and mesothelioma (MESO).

B. **PI3 Expression:** A forest plot illustrating the impact of PI3 expression on OS. Elevated PI3 expression is associated with worse prognosis in glioblastoma and lower-grade glioma (GBMLGG), kidney renal clear cell carcinoma (KIRC), pan-kidney cohort (KIPAN), acute lymphoblastic leukemia (ALL), LAML, OV, pancreatic adenocarcinoma (PAAD), LGG, and lung adenocarcinoma (LUAD).

C. **PAEP Expression:** A forest plot depicting the relationship between PAEP expression and OS. High levels of PAEP predict poor prognosis in PAAD, lung squamous cell carcinoma (LUSC), GBM, head and neck squamous cell carcinoma (HNSC), cervical squamous cell carcinoma and endocervical adenocarcinoma (CESC), uveal melanoma (UVM), and skin cutaneous melanoma (SKCM).

D. **IL27RA Expression:** A forest plot showing the association between IL27RA expression and OS. Increased IL27RA expression is linked to poorer prognosis in GBMLGG, ACC, LAML, ALL, and LGG.

E. **OBP2A Expression:** A forest plot illustrating the impact of OBP2A expression on OS. High OBP2A expression is associated with poor prognosis in KIPAN, KIRC, and mesothelioma (MESO).

F. **PDGFRA Expression:** A forest plot depicting the relationship between PDGFRA expression and OS. Elevated PDGFRA expression predicts worse prognosis in KIPAN, KIRC, and MESO.

G. **FOS Expression:** A forest plot showing the association between FOS expression and OS. High expression of FOS is linked to poor prognosis in ACC, LGG, and GBM.

H. **LRP1 Expression:** A forest plot illustrating the impact of LRP1 expression on OS. Increased LRP1 expression is associated with poorer prognosis in KIPAN, bladder urothelial carcinoma (BLCA), KIRC, LGG, and thyroid carcinoma (THCA).

I. **FGF7 Expression:** A forest plot depicting the relationship between FGF7 expression and OS. Elevated FGF7 expression predicts poor prognosis in PAAD, stomach and esophageal carcinoma (STES), SKCM, LGG, BLCA, STAD, THCA, and KIRP. These findings highlight the significant prognostic value of these IRGs across multiple cancer types, underscoring their potential as biomarkers and therapeutic targets in oncology.

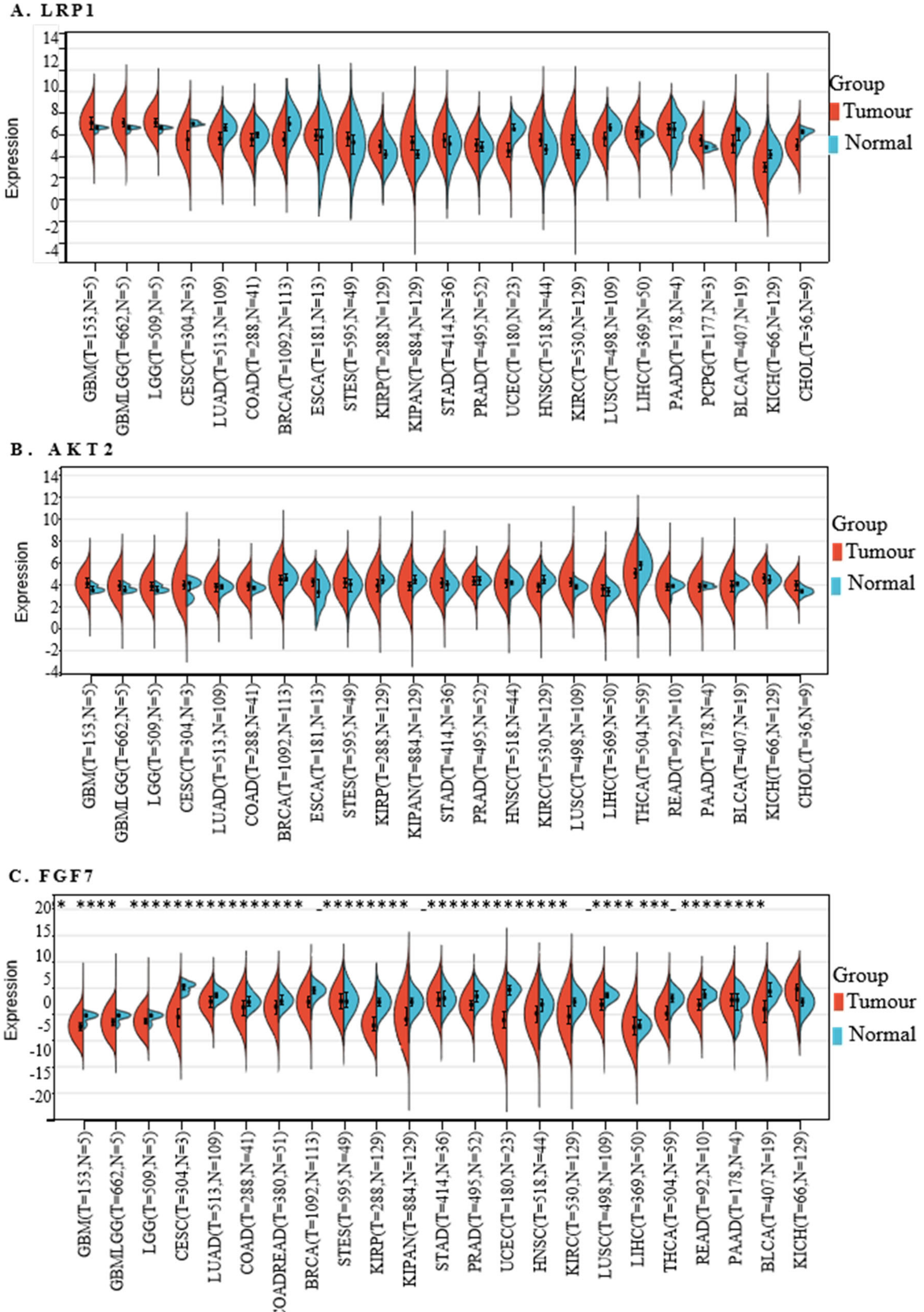
A. LRP1
B. AKT2
C. FGF7
Expression
Group
Tumour
Normal
GBM(T=153,N=5)
GBMLGG(T=662,N=5)
LGG(T=509,N=5)
CESC(T=304,N=3)
LUAD(T=513,N=109)
COAD(T=288,N=41)
COADREAD(T=380,N=51)
BRCA(T=1092,N=113)
ESCA(T=181,N=13)
STES(T=595,N=49)
KIRP(T=288,N=129)
KIPAN(T=884,N=129)
STAD(T=414,N=36)
PRAD(T=495,N=52)
UCEC(T=180,N=23)
HNSC(T=518,N=44)
KIRC(T=530,N=129)
LUSC(T=498,N=109)
LIHC(T=369,N=50)
THCA(T=504,N=59)
READ(T=92,N=10)
PAAD(T=178,N=4)
PCPG(T=177,N=3)
BLCA(T=407,N=19)
KICH(T=66,N=129)
CHOL(T=36,N=9)

D. FOS

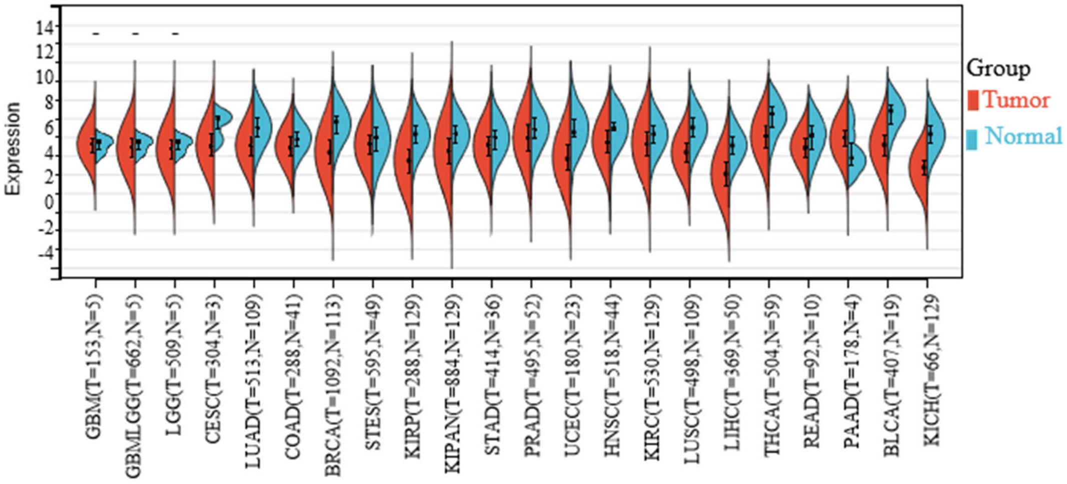

E. IL27RA

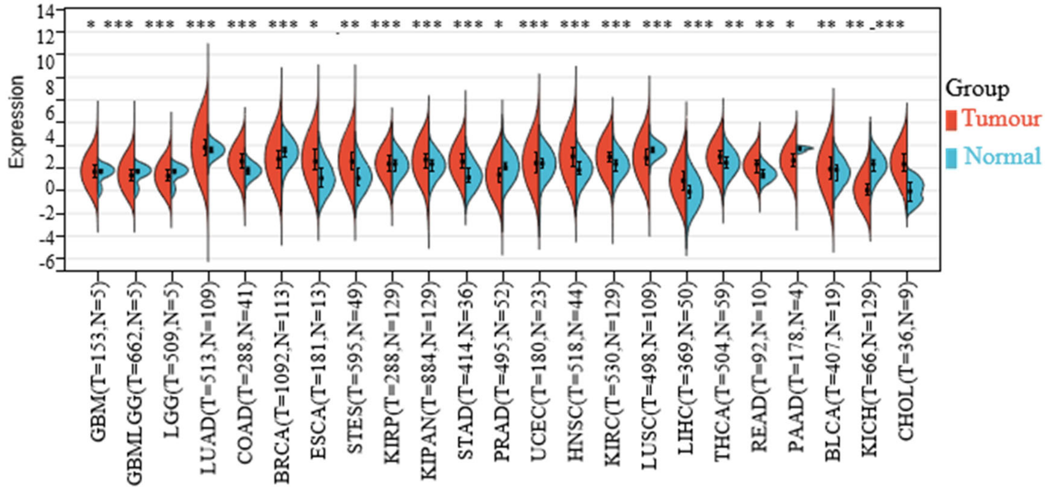

F. OBP2A

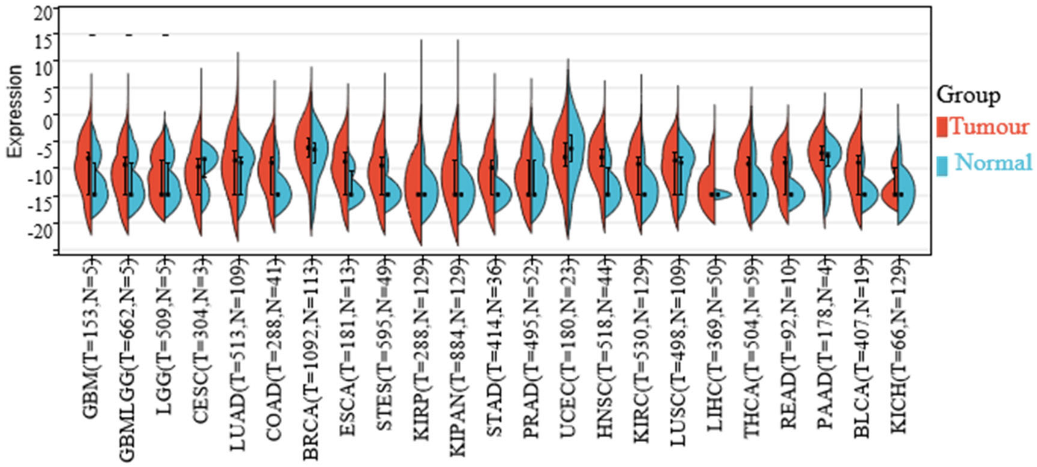

G . P A E P

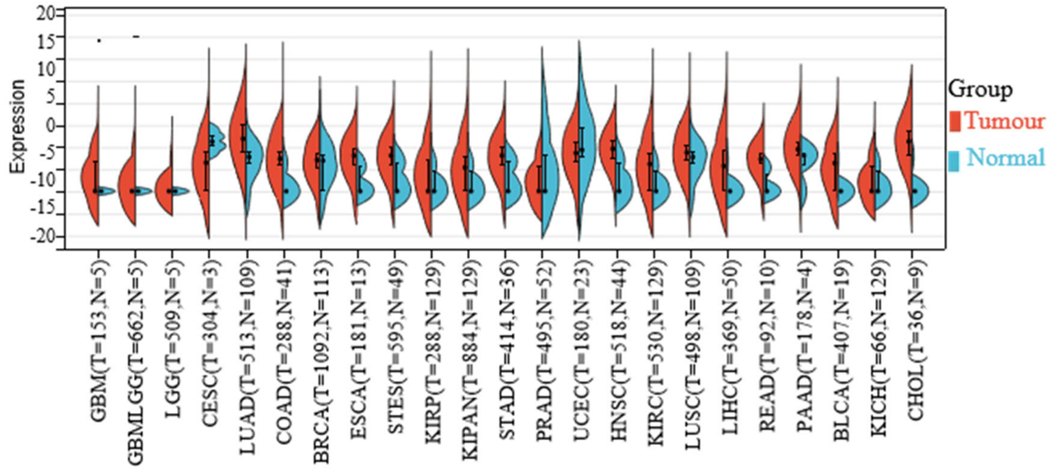

H. PDGFRA

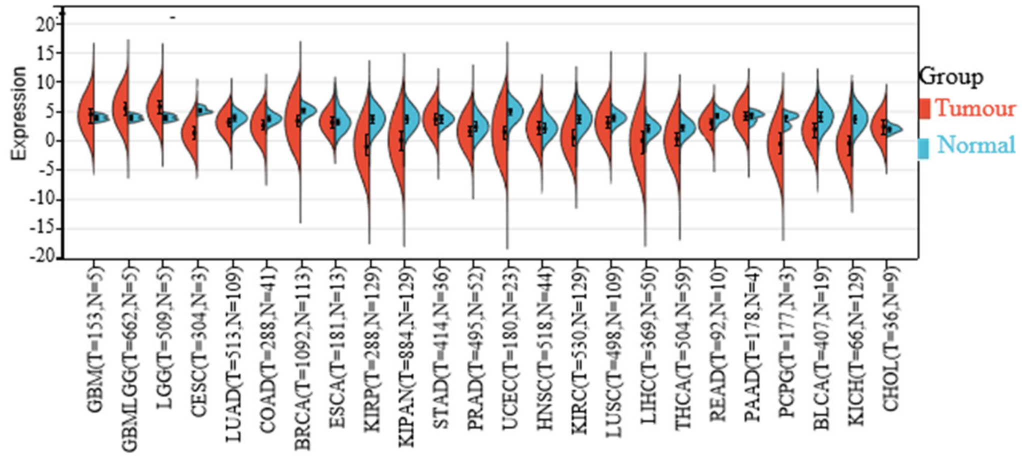

I. PI3

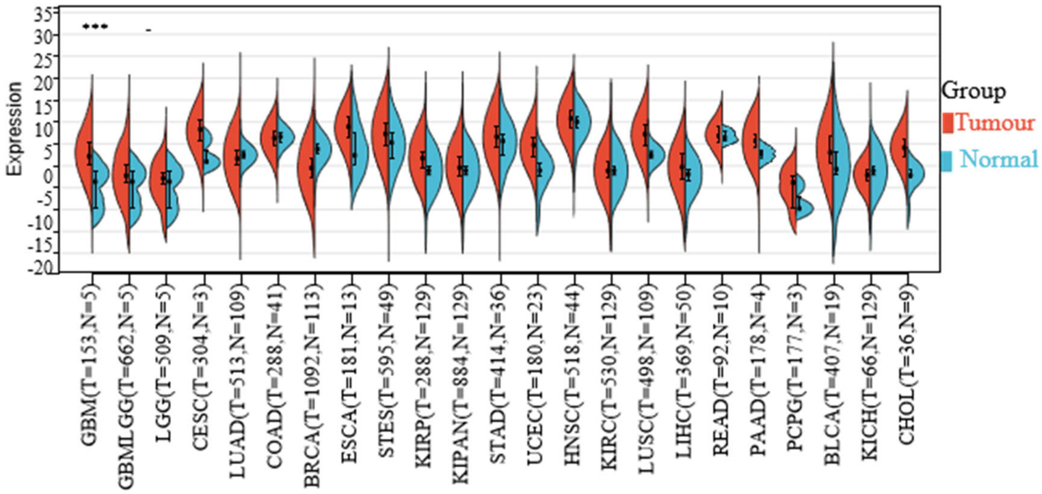

Supplementary Figure S5: The Expression of 9 IRGs Across a Variety of Cancers: illustrates the differential expression of nine immune-related genes (IRGs) across twenty different cancer types compared to their normal tissue counterparts, analyzed using data from The Cancer Genome Atlas

(TCGA) database. Statistical significance is indicated as follows: $P < 0.05$; $P < 0.01$; *$P < 0.001$. These analyses underscore the unique expression profiles of LRP1, AKT2, FGF7, FOS, IL27RA, OBP2A, PAEP, PDGFRA, and PI3 across multiple cancer types, providing insights into their potential roles and importance in cancer biology.

A. **LRP1:** The differential expression of LRP1 in twenty cancer types versus normal tissues, showcasing the variations in its expression levels.
B. **AKT2:** The differential expression of AKT2 across twenty cancer types compared to their normal counterparts, indicating significant differences in expression.
C. **FGF7:** The differential expression of FGF7 in twenty different cancer types relative to normal tissues, revealing its distinct expression patterns.
D. **FOS:** The differential expression of FOS between cancerous and normal tissues in twenty cancer types, highlighting its variable expression.
E. **IL27RA:** The differential expression of IL27RA across twenty cancer types and their normal tissue counterparts, demonstrating significant differences.
F. **OBP2A:** The differential expression of OBP2A in twenty cancer types compared to normal tissues, indicating notable expression variations.
G. **PAEP:** The differential expression of PAEP across twenty cancer types and normal tissues, revealing significant expression differences.
H. **PDGFRA:** The differential expression of PDGFRA between twenty cancer types and their normal counterparts, showcasing distinct expression patterns.
I. **PI3:** The differential expression of PI3 in twenty cancer types versus normal tissues, highlighting significant variations in expression levels.

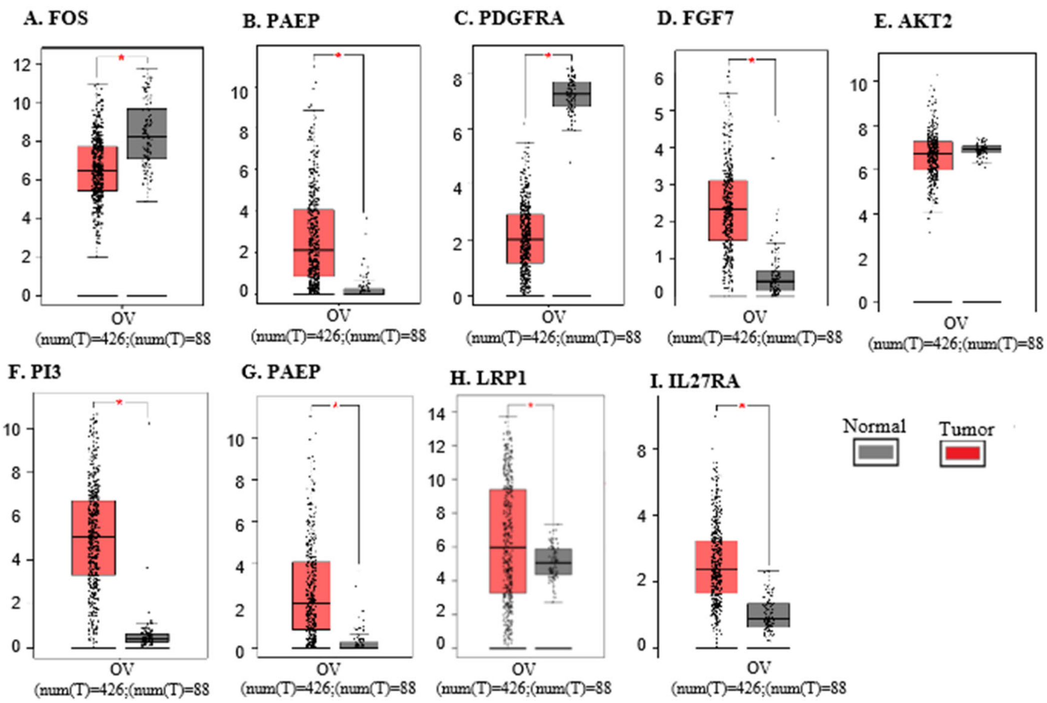

Supplementary Figure S6: Transcriptional Expression Level and Prognostic Value of Differentially Expressed Prognostic Immune-Related Genes in Ovarian Cancer: illustrates the transcriptional expression levels of various prognostic immune-related genes in ovarian cancer compared to normal tissues, using data from the GEPIA database. Each subfigure highlights the differences in expression and their potential prognostic significance.

A. **FOS:** Transcriptional expression levels of FOS in ovarian cancer compared to normal tissues, as analyzed by the GEPIA database.

B. **PAEP:** Transcriptional expression levels of PAEP in ovarian cancer versus normal tissues, showing significant differences as per the GEPIA database.

C. **PDGFRA:** Transcriptional expression levels of PDGFRA in ovarian cancer compared to normal tissues, highlighting its differential expression in the GEPIA database.

D. **FGF7:** Transcriptional expression levels of FGF7 in ovarian cancer versus normal tissues, as depicted in the GEPIA database.

E. **AKT2:** Transcriptional expression levels of AKT2 in ovarian cancer compared to normal tissues, illustrating notable expression differences as per the GEPIA database.

F. **PI3:** Transcriptional expression levels of PI3 in ovarian cancer versus normal tissues, revealing significant variations in the GEPIA database.

G. **PAEP:** Transcriptional expression levels of PAEP in ovarian cancer compared to normal tissues, indicating its prognostic significance in the GEPIA database.

H. **LRP1:** Transcriptional expression levels of LRP1 in ovarian cancer versus normal tissues, as analyzed by the GEPIA database.

I. **IL27RA:** Transcriptional expression levels of IL27RA in ovarian cancer compared to normal tissues, demonstrating differential expression in the GEPIA database.

These analyses provide insights into the transcriptional expression profiles and prognostic values of FOS, PAEP, PDGFRA, FGF7, AKT2, PI3, LRP1, and IL27RA in ovarian cancer, underscoring their potential roles in disease progression and as therapeutic targets.

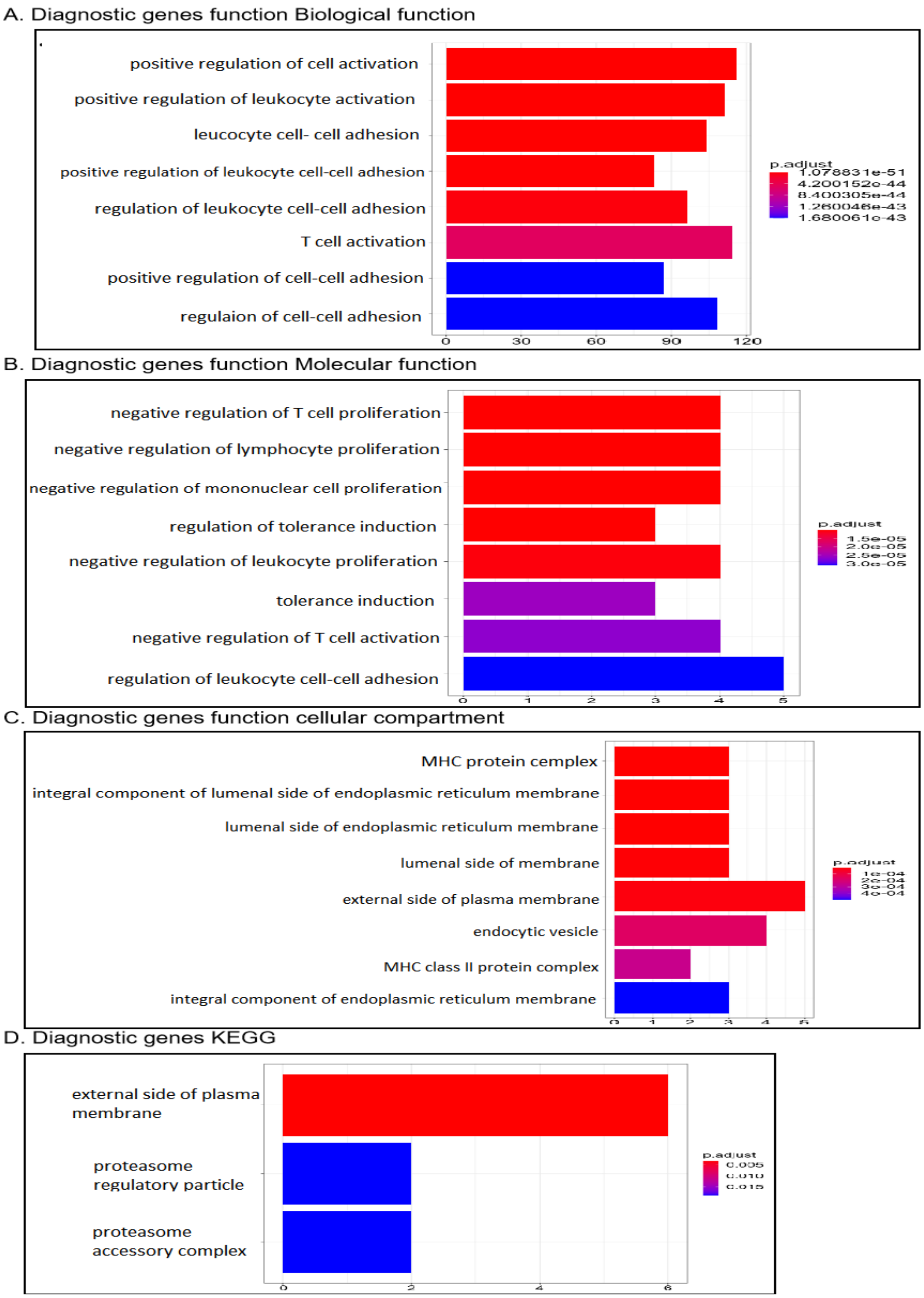

Supplementary Figure S7: GO and KEGG Functional Enrichment Analysis of Immune-Related Pathways Analyzed by GSEA: presents the results of Gene Ontology (GO) and Kyoto Encyclopedia of Genes and Genomes (KEGG) functional enrichment analyses of immune-related pathways using Gene Set Enrichment Analysis (GSEA). The figure is divided into four parts, each highlighting different aspects of the enrichment analysis.

A. **Biological Process (BP):** Gene Ontology analysis of immune-related pathways focusing on biological processes. The bar chart displays the function by gene ratio, indicating the most significantly enriched biological processes involving immune-related genes.

B. **Molecular Function (MF):** Gene Ontology analysis of immune-related pathways focusing on molecular functions. The bar chart illustrates the function by gene ratio, highlighting the key molecular functions that are significantly enriched with immune-related genes.

C. **Cellular Component (CC):** Gene Ontology analysis of immune-related pathways focusing on cellular components. The bar chart shows the function by gene ratio, identifying the cellular components that are most significantly enriched with immune-related genes.

D. **KEGG Pathway Enrichment Analysis:** KEGG pathway enrichment analysis of immune-related genes to identify relevant pathways. The bar chart presents the enriched pathways associated with immune-related genes, providing insights into the biological pathways that these genes are involved in.

These analyses collectively provide a comprehensive overview of the functional roles and pathways associated with immune-related genes, emphasizing their significance in biological processes, molecular functions, cellular components, and specific pathways related to immune response in ovarian cancer.

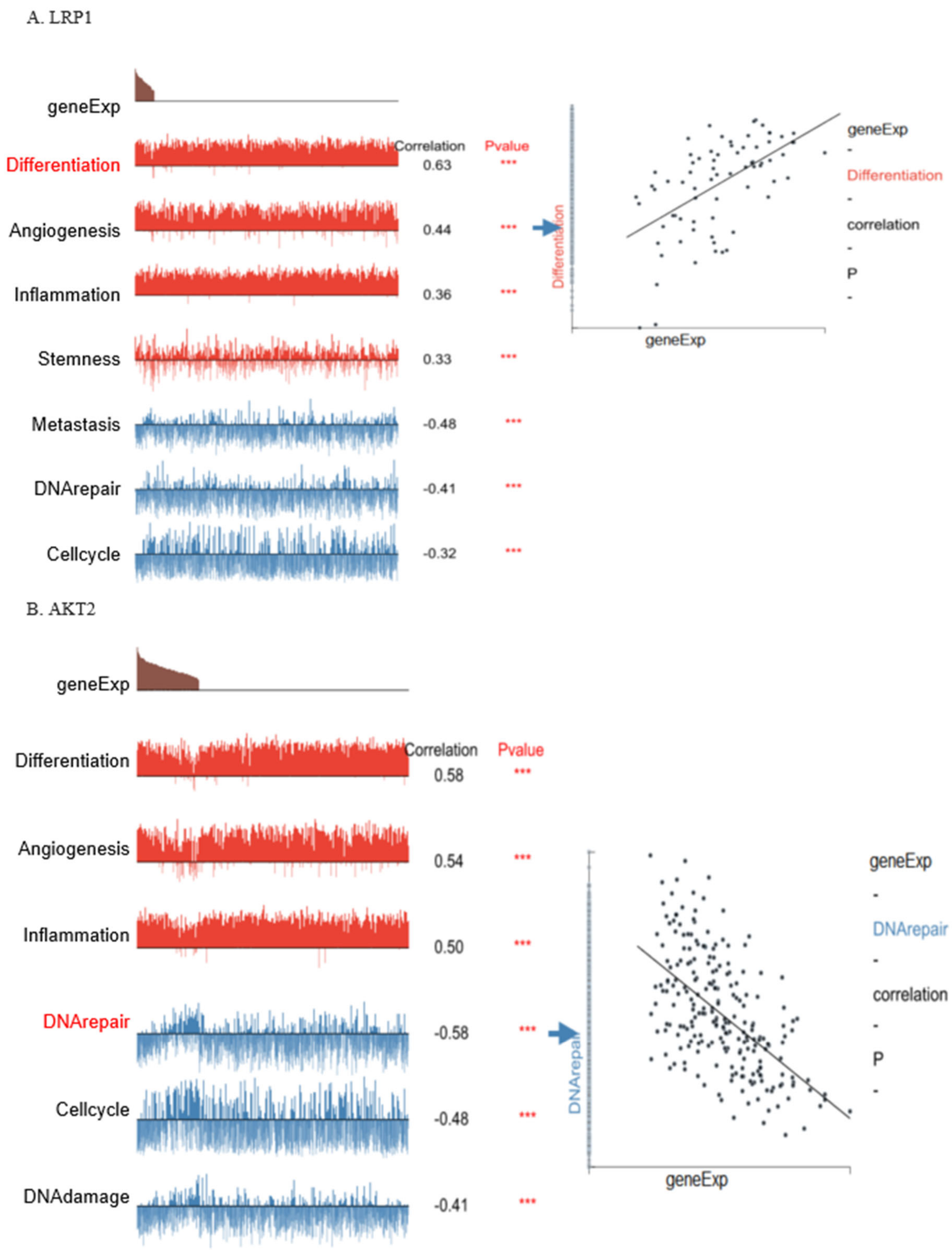

A. LRP1
geneExp
Differentiation
Angiogenesis
Inflammation
Stemness
Metastasis
DNArepair
Cellcycle
Correlation
Pvalue
0.63
0.44
0.36
0.33
-0.48
-0.41
-0.32
***
geneExp
-
Differentiation
-
correlation
-
P
-
Differentiation
geneExp
B. AKT2
geneExp
Differentiation
Angiogenesis
Inflammation
DNArepair
Cellcycle
DNAdamage
Correlation
Pvalue
0.58
0.54
0.50
-0.58
-0.48
-0.41
***
geneExp
-
DNArepair
-
correlation
-
P
-
DNArepair
geneExp

C. FGF7

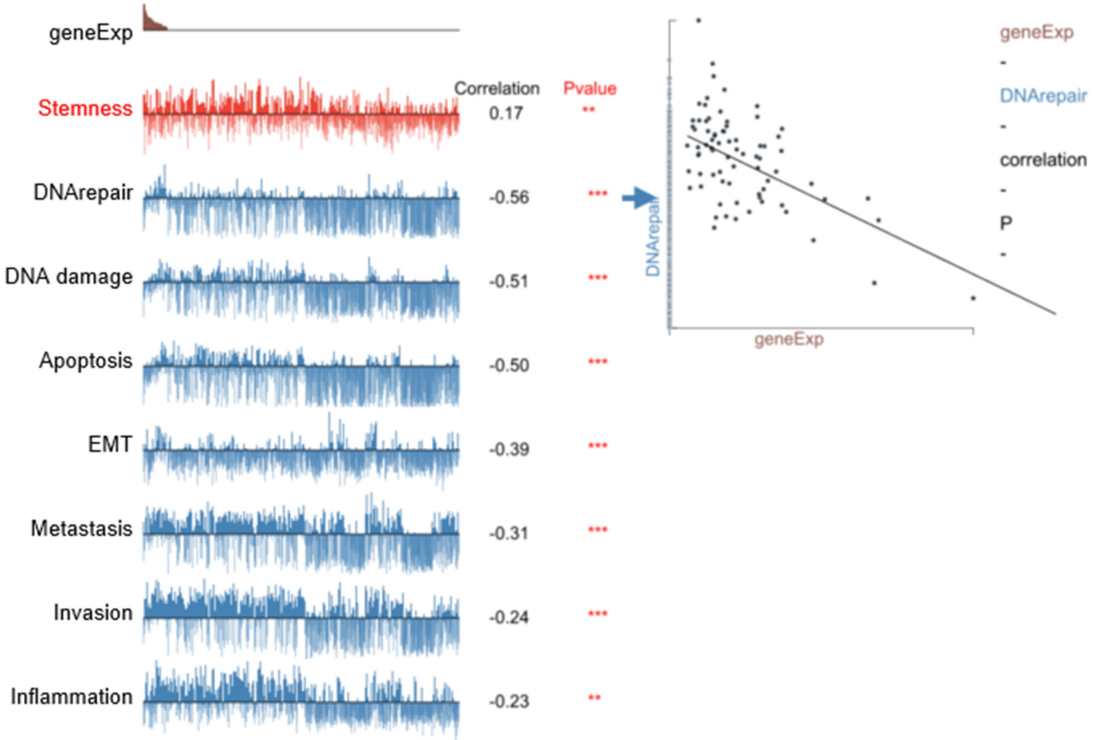

D. OBP2A

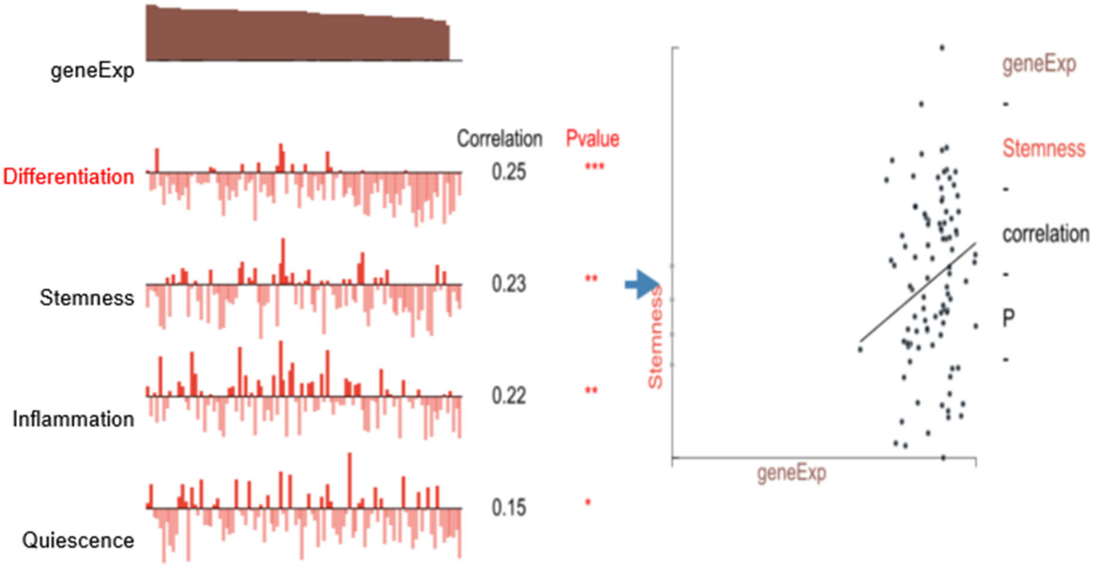

E. IL27RA

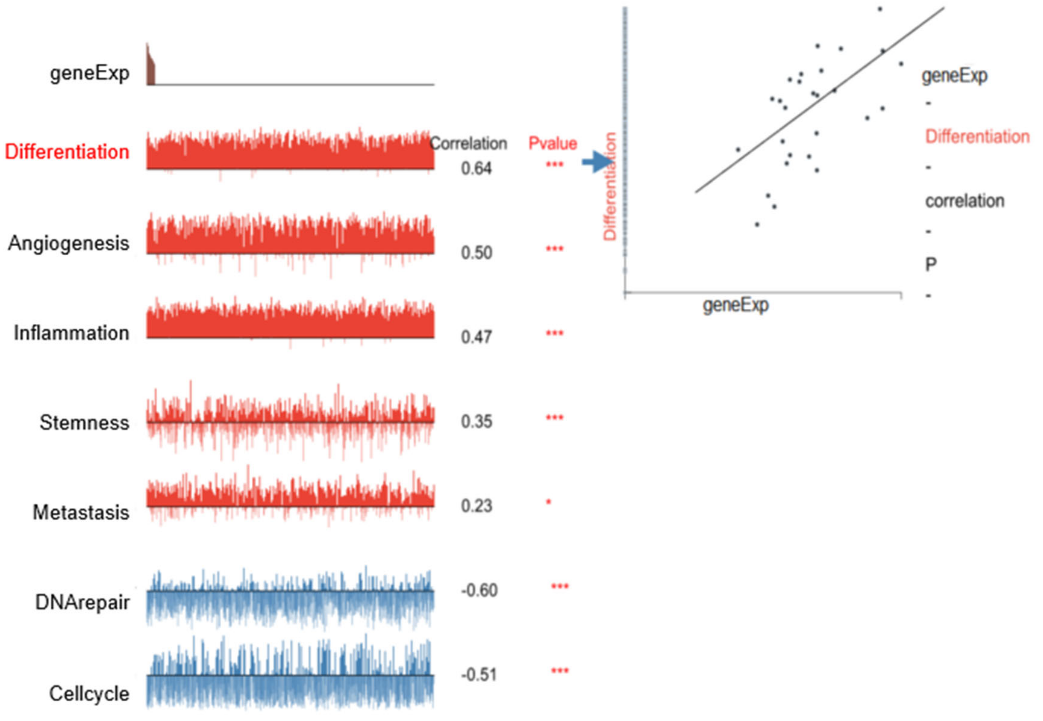

F. OBP2A

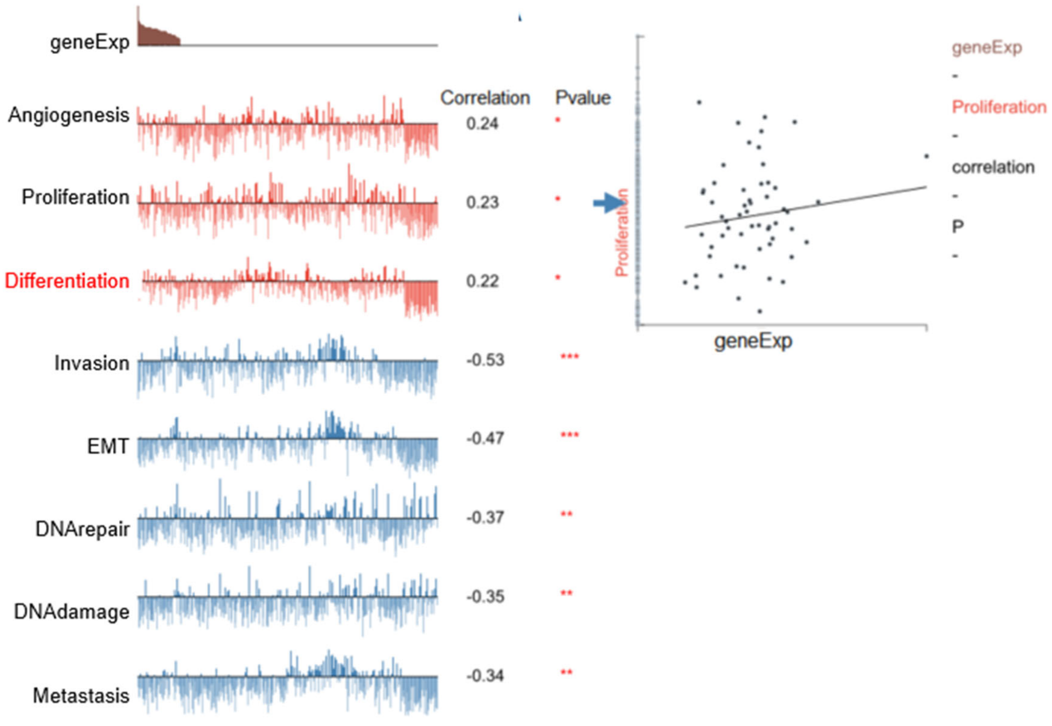

G. PAEP

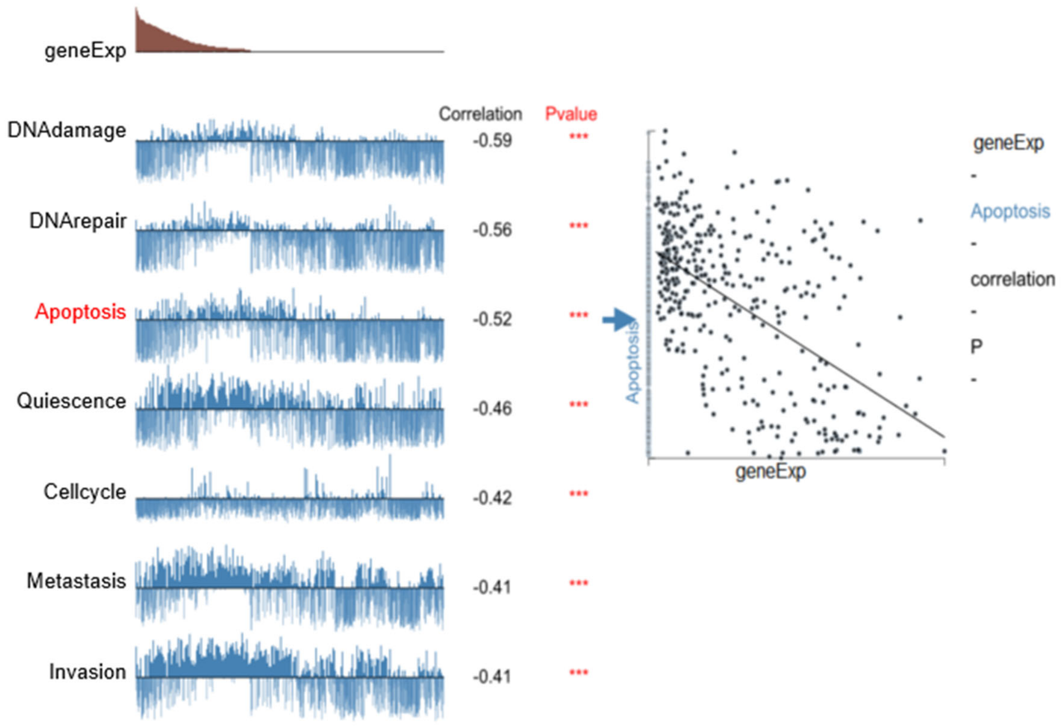

H. PDGFRA

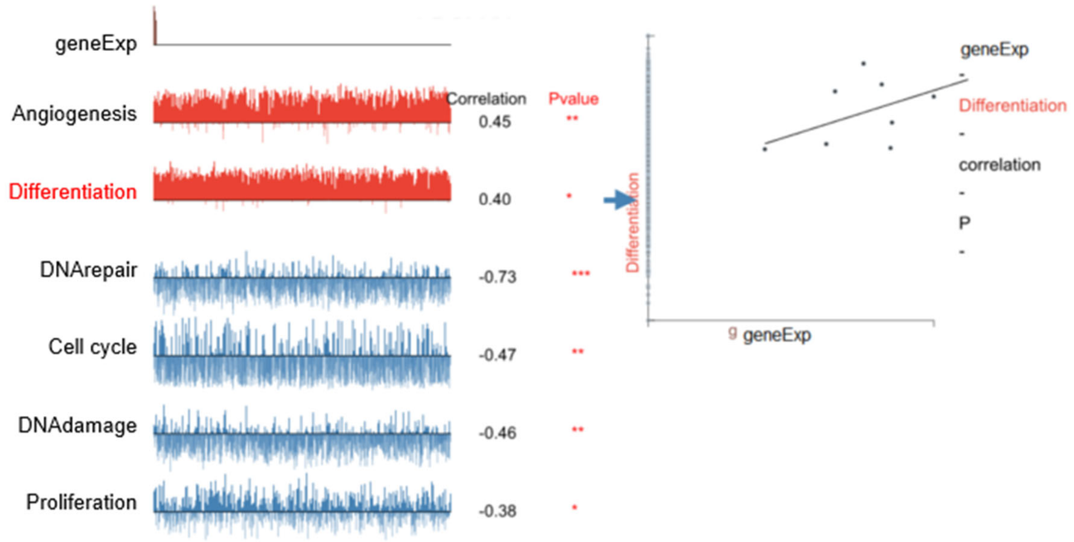

I. PI3

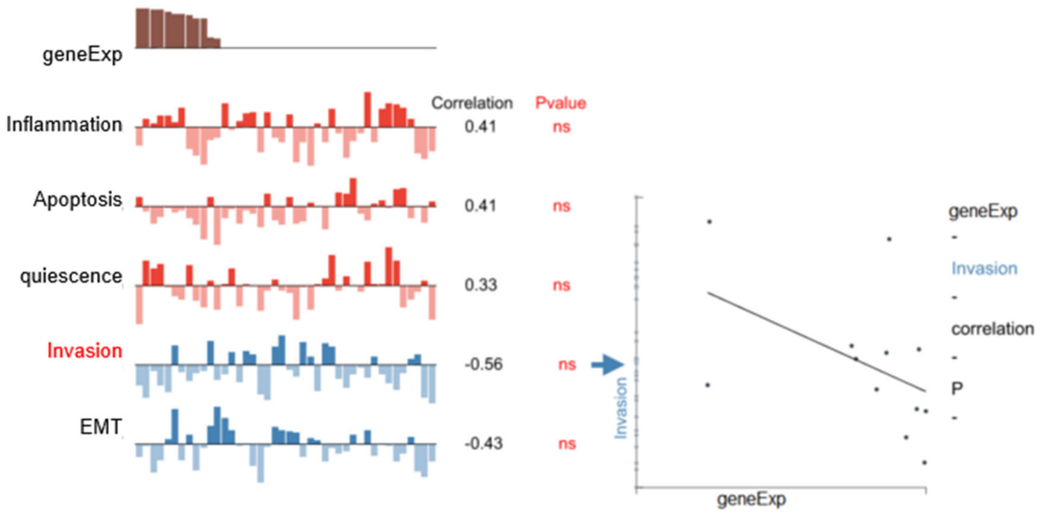

Supplementary Figure S8: Correlation Analysis Between OC Immune-Related Genes and Functional Status of Cancer Cells: illustrates the correlation analysis between ovarian cancer (OC) immune-related genes and various functional statuses of cancer cells through a series of scatter plots. Each scatter plot visualizes the relationship between a specific gene and multiple cancer cell functions.

A. **LRP1:** The scatter plot shows the correlation between LRP1 expression and various functional statuses of cancer cells, including differentiation, angiogenesis, inflammation, DNA repair, and DNA damage.
B. **AKT2:** The scatter plot illustrates the correlation between AKT2 expression and differentiation, angiogenesis, inflammation, stemness, and metastasis in cancer cells.
C. **FGF7:** The scatter plot depicts the correlation between FGF7 expression and stemness, DNA repair, DNA damage, apoptosis, and epithelial-mesenchymal transition (EMT).
D. **FOS:** The scatter plot demonstrates the correlation between FOS expression and differentiation, stemness, inflammation, and quiescence in cancer cells.
E. **IL27RA:** The scatter plot shows the correlation between IL27RA expression and differentiation, angiogenesis, inflammation, stemness, and metastasis.
F. **OBP2A:** The scatter plot illustrates the correlation between OBP2A expression and angiogenesis, proliferation, differentiation, invasion, and EMT.
G. **PAEP:** The scatter plot depicts the correlation between PAEP expression and DNA damage, DNA repair, apoptosis, quiescence, and cell cycle regulation.
H. **PDGFRA:** The scatter plot shows the correlation between PDGFRA expression and angiogenesis, differentiation, DNA repair, cell cycle regulation, and DNA damage.
I. **PI3:** The scatter plot demonstrates the correlation between PI3 expression and inflammation, apoptosis, quiescence, invasion, and EMT.

Legend: *** p <= 0.001; ** p <= 0.01; p <= 0.05; ns. p > 0.05; Grey points were not considered to compute the correlations. These scatter plots highlight the diverse roles and functional impacts of each gene within the context of cancer cell behavior, providing insights into their potential mechanisms of action in ovarian cancer.

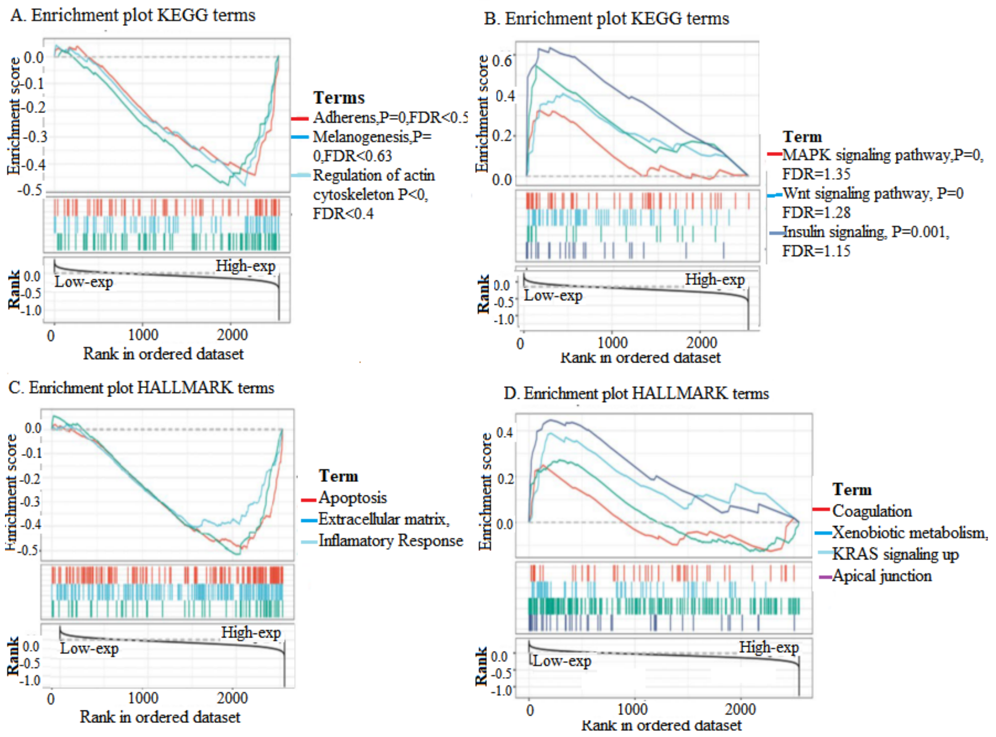

Supplementary Figure S9: Biofunctional Analysis of LRP1 in Ovarian Cancer (OC): presents the biofunctional analysis of LRP1 in ovarian cancer, highlighting the significant pathways and biological processes associated with its expression.

A. KEGG Terms: The KEGG analysis reveals the top three negatively and positively enriched gene sets associated with LRP1 expression in ovarian cancer. Top Three Negative Enrichment Gene Sets: Adherens junction, Melanogenesis, Regulation of cytoskeleton; Top Three Positive Enrichment Gene Sets: MAPK signaling pathway, WNT signaling pathway, Calcium signaling pathway

B. HALLMARK Terms: The HALLMARK analysis identifies the top three negatively enriched gene sets associated with LRP1 expression. Top Three Negative Enrichment Gene Sets: Apoptosis, Extracellular matrix, and Inflammatory response

C. HALLMARK Terms: The HALLMARK analysis also identifies the top three positively enriched gene sets associated with LRP1 expression. Top Three Positive Enrichment Gene Sets: Coagulation, Xenobiotic metabolism, KRAS signaling up, and Apical junction

D. Association of LRP1 Expression with Pathways and Processes:

The bar chart and scatter plots depict how high expression of LRP1 is associated with various pathways and biological processes.

Negative Association: Adherens junction, Melanogenesis, Regulation of cytoskeleton, Apoptosis, Extracellular matrix, Inflammatory response, Positive Association: MAPK signaling pathway WNT signaling pathway, Calcium signaling pathway, Coagulation, Xenobiotic metabolism, KRAS signaling up and Apical junction. These analyses provide a comprehensive view of how LRP1 expression influences various signaling pathways and biological processes in ovarian cancer, underscoring its potential role in tumour progression and therapeutic targeting.

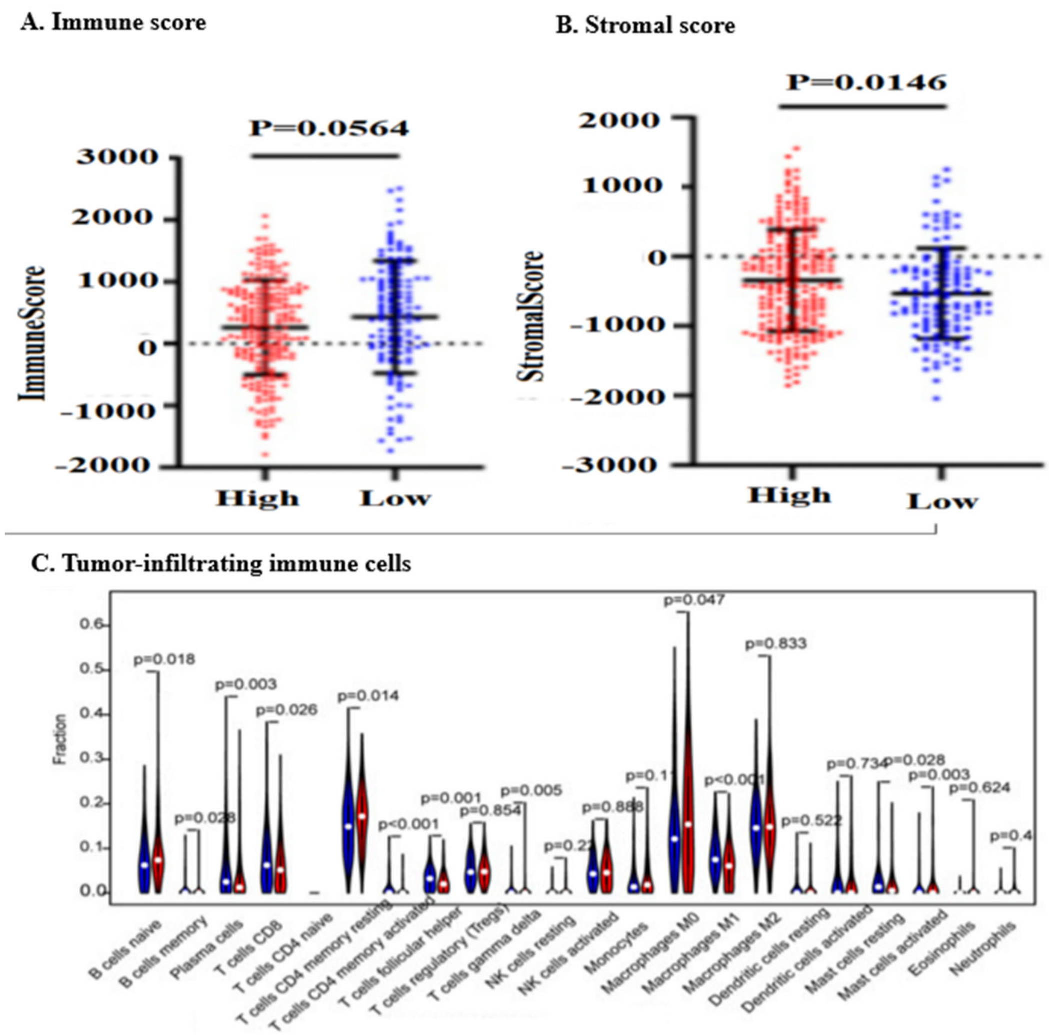

Supplementary Figure S10: Correlation Between mRNA Characteristics and Immune Cell Infiltration: illustrates the relationship between the expression of specific mRNA characteristics and immune cell infiltration in ovarian cancer.

A. **Immune Scores in High- and Low-Risk Groups Using the "ESTIMATE" Algorithm:** This panel displays the immune scores calculated using the "ESTIMATE" algorithm, comparing the high-risk and low-risk groups. The immune scores reflect the overall level of immune cell infiltration within the tumour microenvironment. High-Risk Group: Shows a specific range of immune scores. Low-Risk Group: Displays a different range of immune scores compared to the high-risk group.

B. **Immune Scores in High- and Low-Risk Groups Using the "ESTIMATE" Algorithm:** This panel again calculates the immune scores using the "ESTIMATE" algorithm for a separate set of mRNA characteristics, comparing the high-risk and low-risk groups. High-Risk Group: Shows the immune scores based on the new set of mRNA characteristics. Low-Risk Group: Displays the immune scores for the same set of characteristics in the low-risk group.

C. **Tumour-Infiltrating Immune Cells Abundance in the Two Groups:** This panel discusses the abundance of various tumour-infiltrating immune cells in the high-risk and low-risk groups. The presence and quantity of different types of immune cells within the tumour microenvironment can

influence the tumour's behavior and the patient's prognosis. High-Risk Group: The abundance and types of immune cells present in this group. Low-Risk Group: The comparison of immune cell infiltration in this group.

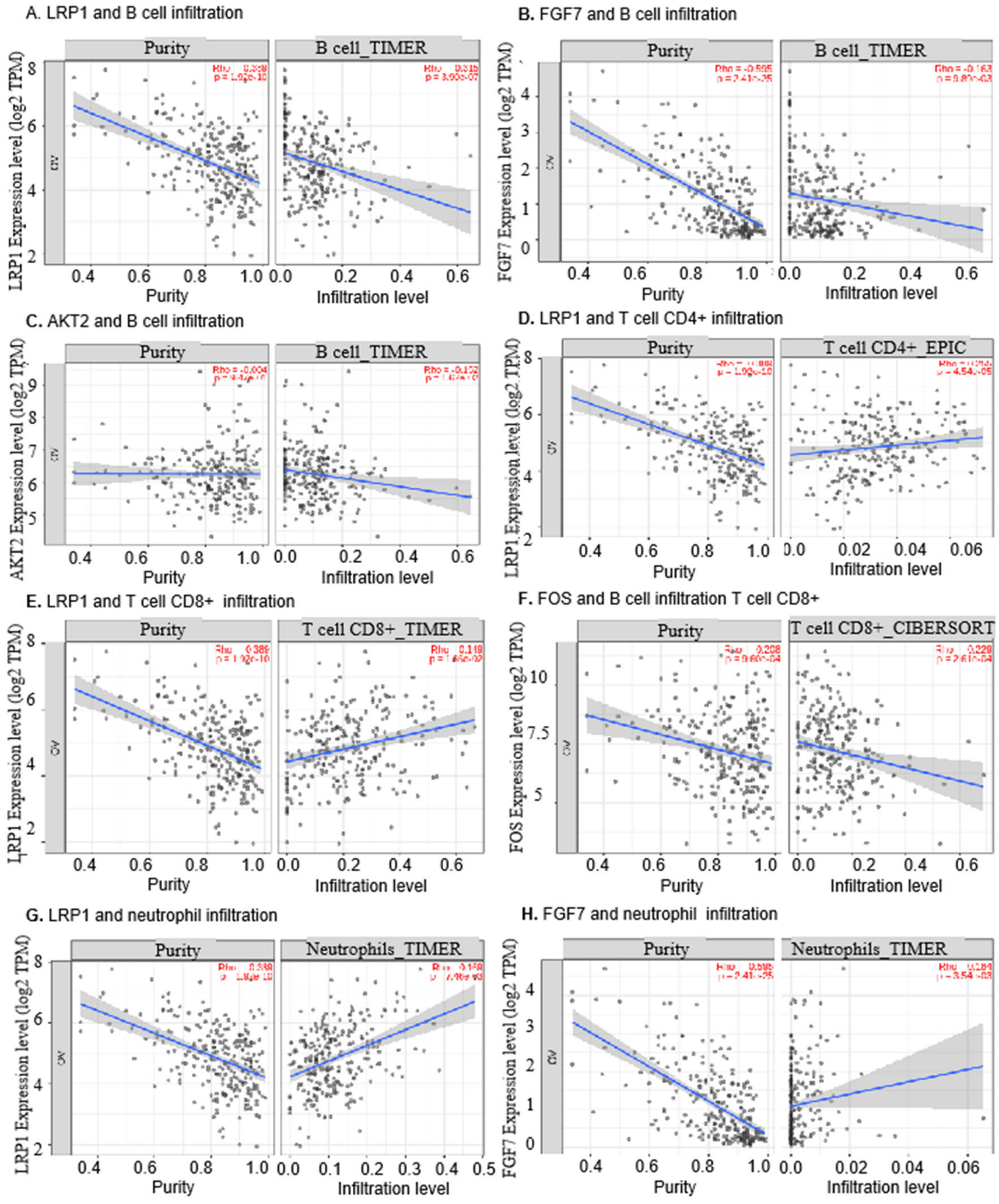

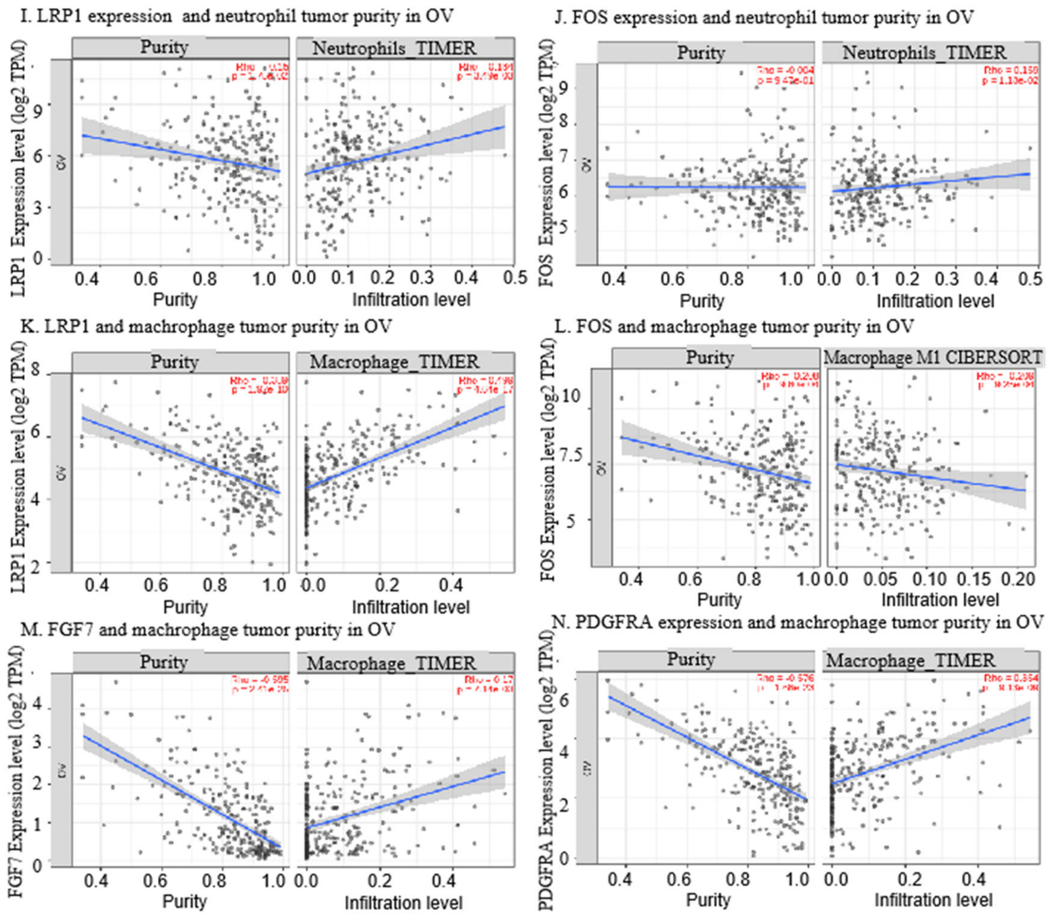

Supplementary Figure S11: Correlation of 9 Immune-Related Genes (IRGs) Expression with Stromal Score in Ovarian Cancer

A. The expression level of LRP1 (Low-Density Lipoprotein Receptor-Related Protein 1) was found to be negatively correlated with the infiltration levels of B cells and tumour purity in ovarian cancer (OC).

B. FGF7 (Fibroblast Growth Factor 7) expression exhibited a negative correlation with the infiltration levels of B cells and tumour purity in ovarian cancer (OC).

C. AKT2 (Protein Kinase B) expression was negatively associated with infiltration levels of B cells and tumour purity in ovarian cancer (OC).

D. LRP1 expression showed a negative correlation with infiltration levels of CD4+ cells and tumour purity in ovarian cancer (OC).

E. LRP1 expression displayed a negative association with infiltration levels of CD8+ cells and tumour purity in ovarian cancer (OC).

F. FOS (FBJ Murine Osteosarcoma Viral Oncogene Homolog) expression was negatively correlated with infiltration levels of CD8+ cells and tumour purity in ovarian cancer (OC).

G. LRP1 expression demonstrated a negative correlation with infiltration levels of neutrophils and tumour purity in ovarian cancer (OC).

H. FGF7 expression exhibited a negative association with infiltration levels of neutrophils and tumour purity in ovarian cancer (OC).

I. LRP1 expression was negatively associated with infiltration levels of neutrophils and tumour purity in ovarian cancer (OC).

J. FOS expression showed a negative correlation with infiltration levels of neutrophils and tumour purity in ovarian cancer (OC).

K. LRP1 expression displayed a negative correlation with infiltration levels of macrophages and tumour purity in ovarian cancer (OC).

L. FOS expression was negatively associated with infiltration levels of macrophages and tumour purity in ovarian cancer (OC).

M. FGF7 expression showed a negative correlation with infiltration levels of macrophages and tumour purity in ovarian cancer (OC).

N. PDGFRA (Platelet-Derived Growth Factor Receptor Alpha) expression exhibited a negative association with infiltration levels of macrophages and tumour purity in ovarian cancer (OC).

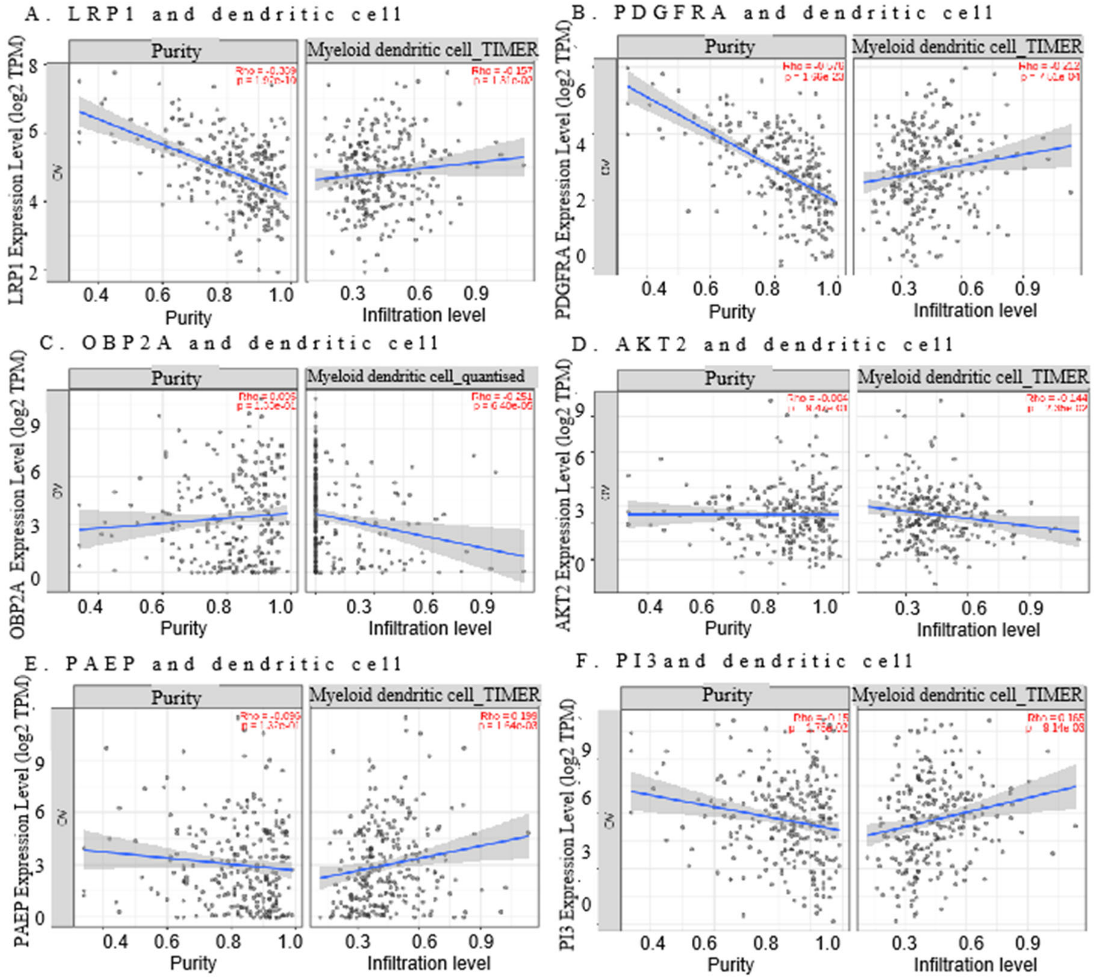

Supplementary Figure S12: Correlation of 9 Immune-Related Genes (IRGs) Expression with Stromal Score and Immune Cell Infiltration in Ovarian Cancer (OC): It depicts the correlation between the expression levels of 9 immune-related genes (IRGs) and stromal score, as well as the association between these genes and immune cell infiltration in ovarian cancer (OC).

A. **LRP1 (Low-Density Lipoprotein Receptor-Related Protein 1)** expression exhibited a negative association with the infiltration levels of myeloid dendritic cells and tumour purity in OC.

B. **PDGFRA (Platelet-Derived Growth Factor Receptor Alpha)** expression was found to be negatively correlated with infiltration levels of myeloid dendritic cells and tumour purity in OC.

C. **OBP2A e**xpression displayed a negative correlation with infiltration levels of myeloid dendritic cells and tumour purity in OC.

D. **AKT2 (Protein Kinase B)** expression showed a negative association with infiltration levels of myeloid dendritic cells and tumour purity in OC.

E. **PAEP (Progestagen-Associated Endometrial Protein)** expression was positively associated with infiltration levels of myeloid dendritic cells and tumour purity in OC.

F. **PI3 (Phosphatidylinositol 3-kinase)** expression exhibited a positive correlation with infiltration levels of myeloid dendritic cells and tumour purity in OC.

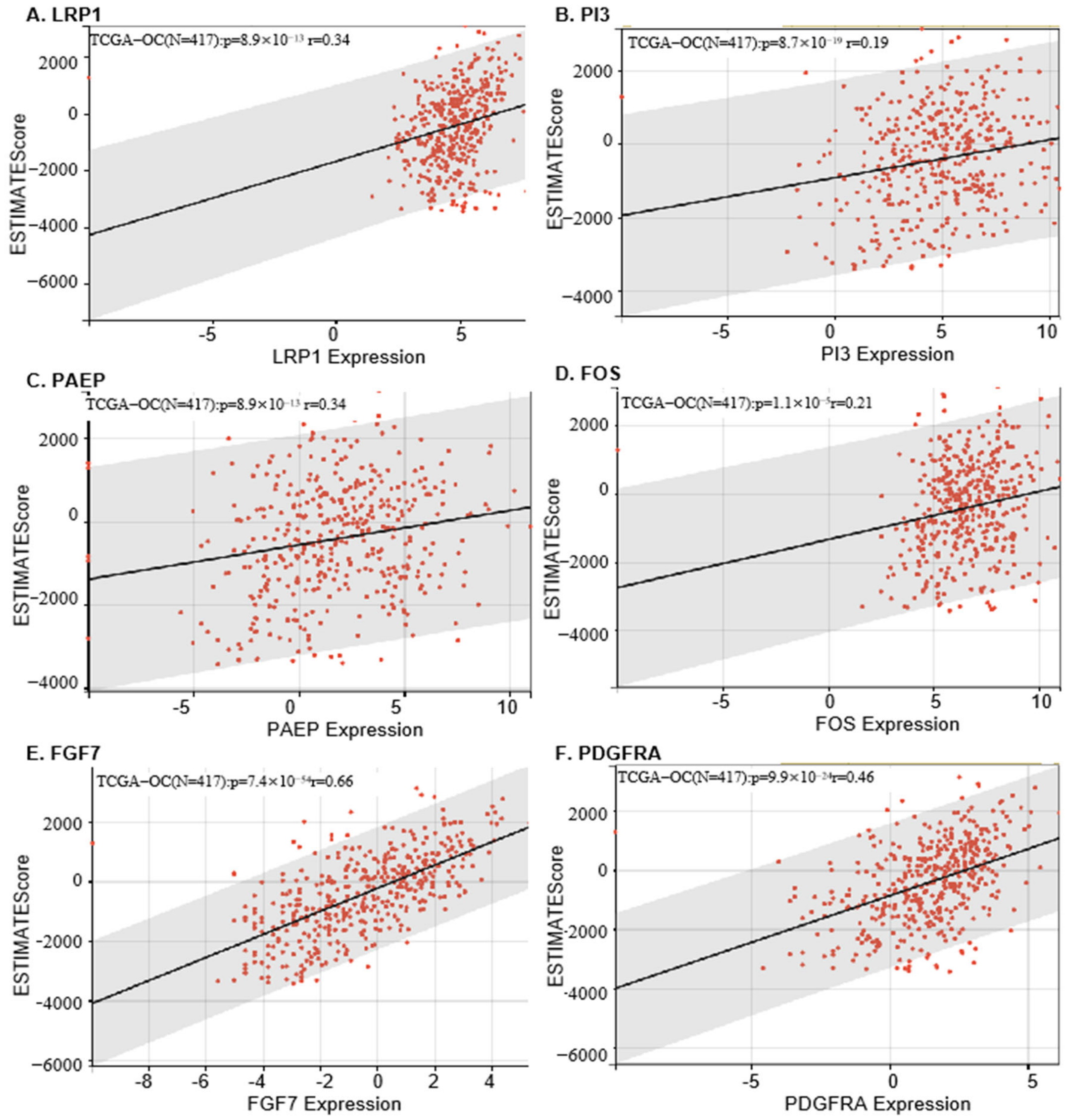

Supplementary Figure S13: Correlation of 9 Immune-Related Genes (IRGs) Expression with Estimate Score in Ovarian Cancer (OC) illustrates the correlation between the expression levels of 9 immune-related genes (IRGs) and Estimate Score, a measure of tumour purity, in ovarian cancer (OC).

A. **LRP1 (Low-Density Lipoprotein Receptor-Related Protein 1)** expression showed a positive relationship with tumour purity in OC.

B. **PI3 (Phosphatidylinositol 3-kinase)** expression exhibited a positive correlation with tumour purity in OC.

C. **PAEP (Progestagen-Associated Endometrial Protein)** expression was positively related to tumour purity in OC.

D. **FOS (FBJ Murine Osteosarcoma Viral Oncogene Homolog)** expression showed a positive relationship with tumour purity in OC.

E. **FGF7 (Fibroblast Growth Factor 7)** expression displayed a positive correlation with tumour purity in OC.

F. **PDGFRA (Platelet-Derived Growth Factor Receptor Alpha)** expression was positively related to tumour purity in OC.

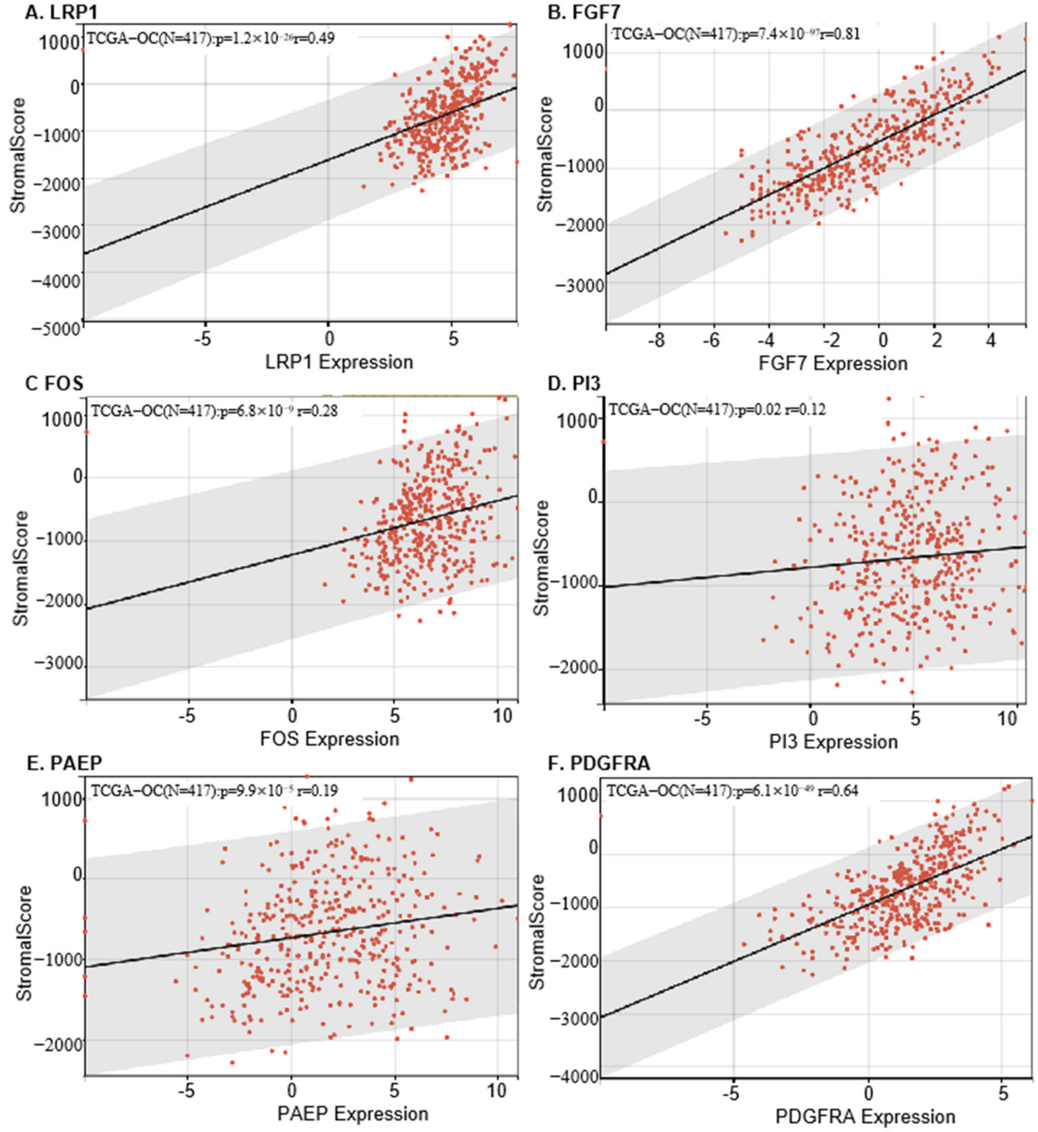

Supplementary Figure S14: Correlation of 9 Immune-Related Genes (IRGs) Expression with Stromal Score in Ovarian Cancer (OC): It depicts the correlation between the expression levels of 9 immune-related genes (IRGs) and Stromal Score, reflecting the stromal component of the tumour microenvironment, in ovarian cancer (OC).

A. **LRP1 (Low-Density Lipoprotein Receptor-Related Protein 1)** expression exhibited a positive association with tumour purity in OC.

B. **FGF7 (Fibroblast Growth Factor 7)** expression showed a positive correlation with tumour purity in OC.

C. **FOS (FBJ Murine Osteosarcoma Viral Oncogene Homolog)** expression was positively associated with tumour purity in OC.

D. **PI3 (Phosphatidylinositol 3-kinase)** expression exhibited a positive relationship with tumour purity in OC.

E. **PAEP (Progestagen-Associated Endometrial Protein)** expression was positively associated with tumour purity in OC.

F. **PDGFRA (Platelet-Derived Growth Factor Receptor Alpha)** expression displayed a positive correlation with tumour purity in OC.

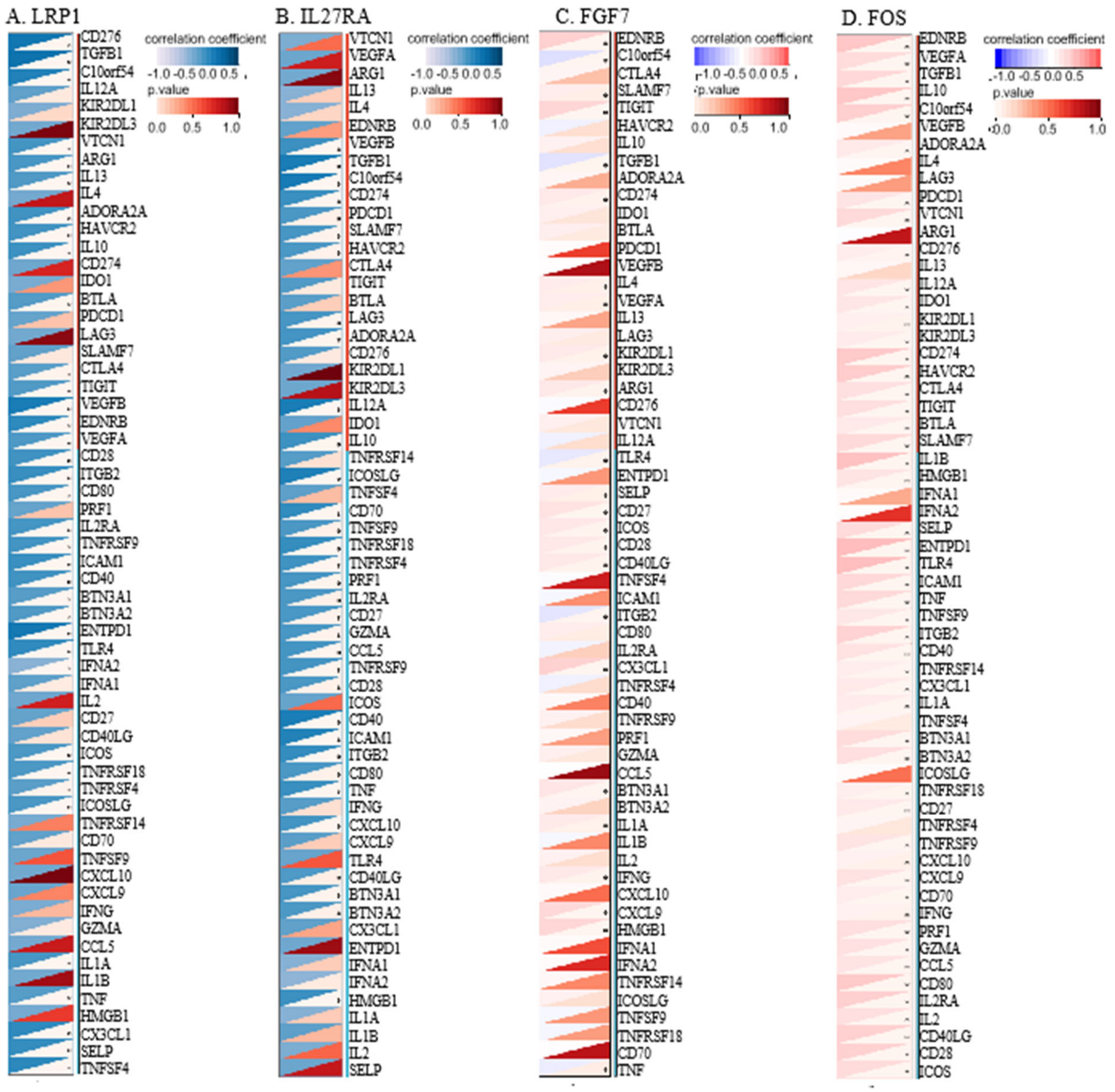
A. LRP1
CD276
TGFB1
C10orf54
IL12A
KIR2DL1
KIR2DL3
VTCN1
ARG1
IL13
IL4
ADORA2A
HAVCR2
IL10
CD274
IDO1
BTLA
PDCD1
LAG3
SLAMF7
CTLA4
TIGIT
VEGFB
EDNRB
VEGFA
CD28
ITGB2
CD80
PRF1
IL2RA
TNFRSF9
ICAM1
CD40
BTN3A1
BTN3A2
ENTPD1
TLR4
IFNA2
IFNA1
IL2
CD27
CD40LG
ICOS
TNFRSF18
TNFRSF4
ICOSLG
TNFRSF14
CD70
TNFSF9
CXCL10
CXCL9
IFNG
GZMA
CCL5
IL1A
IL1B
TNF
HMGB1
CX3CL1
SELP
TNFSF4
correlation coefficient
-1.0 -0.5 0.0 0.5
p.value
0.0 0.5 1.0
B. IL27RA
VTCN1
VEGFA
ARG1
IL13
IL4
EDNRB
VEGFB
TGFB1
C10orf54
CD274
PDCD1
SLAMF7
HAVCR2
CTLA4
TIGIT
BTLA
LAG3
ADORA2A
CD276
KIR2DL1
KIR2DL3
IL12A
IDO1
IL10
TNFRSF14
ICOSLG
TNFSF4
CD70
TNFSF9
TNFRSF18
TNFRSF4
PRF1
IL2RA
CD27
GZMA
CCL5
TNFRSF9
CD28
ICOS
CD40
ICAM1
ITGB2
CD80
TNF
IFNG
CXCL10
CXCL9
TLR4
CD40LG
BTN3A1
BTN3A2
CX3CL1
ENTPD1
IFNA1
IFNA2
HMGB1
IL1A
IL1B
IL2
SELP
correlation coefficient
-1.0 -0.5 0.0 0.5
p.value
0.0 0.5 1.0
C. FGF7
EDNRB
C10orf54
CTLA4
SLAMF7
TIGIT
HAVCR2
IL10
TGFB1
ADORA2A
CD274
IDO1
BTLA
PDCD1
VEGFB
IL4
VEGFA
IL13
LAG3
KIR2DL1
KIR2DL3
ARG1
CD276
VTCN1
IL12A
TLR4
ENTPD1
SELP
CD27
ICOS
CD28
CD40LG
TNFSF4
ICAM1
ITGB2
CD80
IL2RA
CX3CL1
TNFRSF4
CD40
TNFRSF9
PRF1
GZMA
CCL5
BTN3A1
BTN3A2
IL1A
IL1B
IL2
IFNG
CXCL10
CXCL9
HMGB1
IFNA1
IFNA2
TNFRSF14
ICOSLG
TNFSF9
TNFRSF18
CD70
TNF
correlation coefficient
-1.0 -0.5 0.0 0.5
p.value
0.0 0.5 1.0
D. FOS
EDNRB
VEGFA
TGFB1
IL10
C10orf54
VEGFB
ADORA2A
IL4
LAG3
PDCD1
VTCN1
ARG1
CD276
IL13
IL12A
IDO1
KIR2DL1
KIR2DL3
CD274
HAVCR2
CTLA4
TIGIT
BTLA
SLAMF7
IL1B
HMGB1
IFNA1
IFNA2
SELP
ENTPD1
TLR4
ICAM1
TNF
TNFSF9
ITGB2
CD40
TNFRSF14
CX3CL1
IL1A
TNFSF4
BTN3A1
BTN3A2
ICOSLG
TNFRSF18
CD27
TNFRSF4
TNFRSF9
CXCL10
CXCL9
CD70
IFNG
PRF1
GZMA
CCL5
CD80
IL2RA
IL2
CD40LG
CD28
ICOS
correlation coefficient
-1.0 -0.5 0.0 0.5
p.value
0.0 0.5 1.0

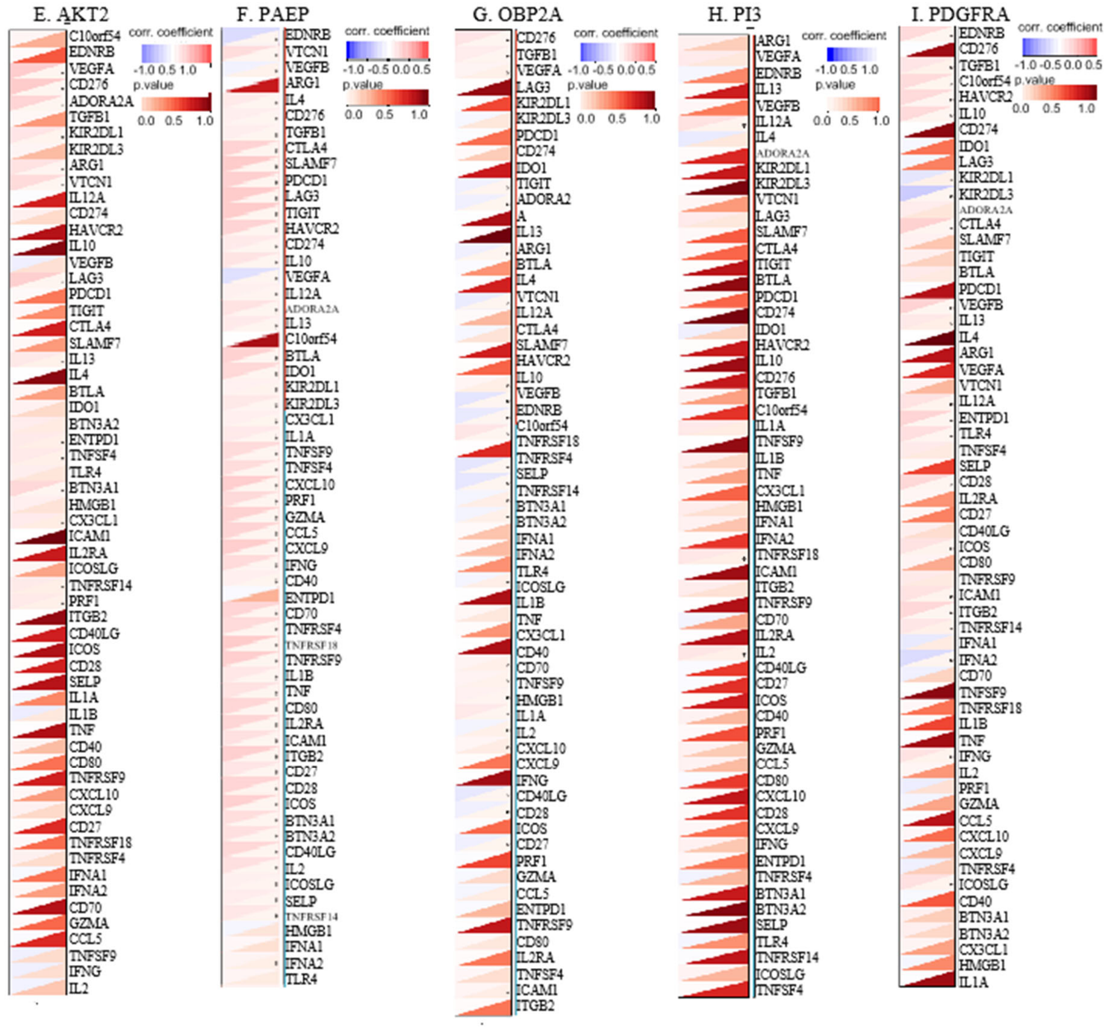

Supplementary Figure S15: Pearson Correlation Analysis between 9 Immune-Related Genes (IRGs) Expression and Immune Checkpoint Genes in Ovarian Cancer (OC): the results of Pearson correlation analysis between the expression levels of 9 immune-related genes (IRGs) and immune checkpoint genes in ovarian cancer (OC), providing insights into potential regulatory relationships within the tumour microenvironment.

A. The expression of LRP1 (Low-Density Lipoprotein Receptor-Related Protein 1) was found to be positively correlated with various immune checkpoint markers in OC. Increased LRP1 expression was positively correlated with CD274 (PD-L1), TGFB1, C10orf54, VCTN1, ARG1, IL13, ADORA2A, HAVCR2 (TIM-3), IL10, BTLA, CTLA4, TIGIT, VEGFB, CD28, ITGB2, CD80, IL2RA, and CD40 in ovarian cancer.

B. The expression of IL27RA (Interleukin 27 Receptor Alpha) exhibited a positive correlation with immune checkpoint markers in OC. Increased IL27RA expression was positively correlated with VEGFB, TGFB1, C10orf54, VCTN1, CD274, PDCD1 (PD-1), LAG3, IL12A, CD70, ICOSLG, PRF1, IL2RA, and CD27.

C. FGF7 (Fibroblast Growth Factor 7) expression showed a positive correlation with immune checkpoint markers in OC. Increased FGF7 expression was positively correlated with ENDRB, C10orf54, SLAMF, TIGIT, TGFB1, CD274, IL4, TLR4, ENTPD1, SELP, CD27, and ICOS.

D. FOS (FBJ Murine Osteosarcoma Viral Oncogene Homolog) expression was positively correlated with immune checkpoint markers in OC. Increased FOS expression was positively correlated with ENDRA, VEGFA, C10orf54, TGFB1, IL10, ADORA2A, PDCD1, IL1B, HMGB1, ENTPD1, SELP, and TNF.

E. AKT2 (Protein Kinase B) expression exhibited a positive correlation with immune checkpoint markers in OC. Increased AKT2 expression was positively correlated with VEGFB, CD274, TGFB1, KIR2DL1, ARG1, ADORA2A, LAG3, BTLA, IL4, PRF1, and TLR4.

F. PAEP (Progestagen-Associated Endometrial Protein) expression showed a positive correlation with immune checkpoint markers in OC. Increased PAEP expression was positively correlated with ENDRB, VTCN1, VEGFB, IL4, CD276, TGFB1, CTLA4, CXCL1, IL1A, TNFSF9, TNFSF4, and CXCL10.

G. OBP2A expression exhibited a positive correlation with immune checkpoint markers in OC. Increased OBP2A expression was positively correlated with CD274, TGFB1, VEGFA, TIGIT, IL12A, ADORA2A, TNFRSF18, SELP, and BTN3A2.

H. PI3 (Phosphatidylinositol 3-kinase) expression was positively correlated with immune checkpoint markers in OC. Increased PI3 expression was positively correlated with IL12A, TNFRSF9, and IL2.

I. PDGFRA (Platelet-Derived Growth Factor Receptor Alpha) expression showed a positive correlation with immune checkpoint markers in OC. Increased PDGFRA expression was positively correlated with ENDRB, TGFB1, C10orf54, HAVCR2, IL10, CTLA4, ENTPD1, TLR4, CD28, and ICOS. *Indicates a p-value < 0.05, suggesting statistical significance.

A．OC

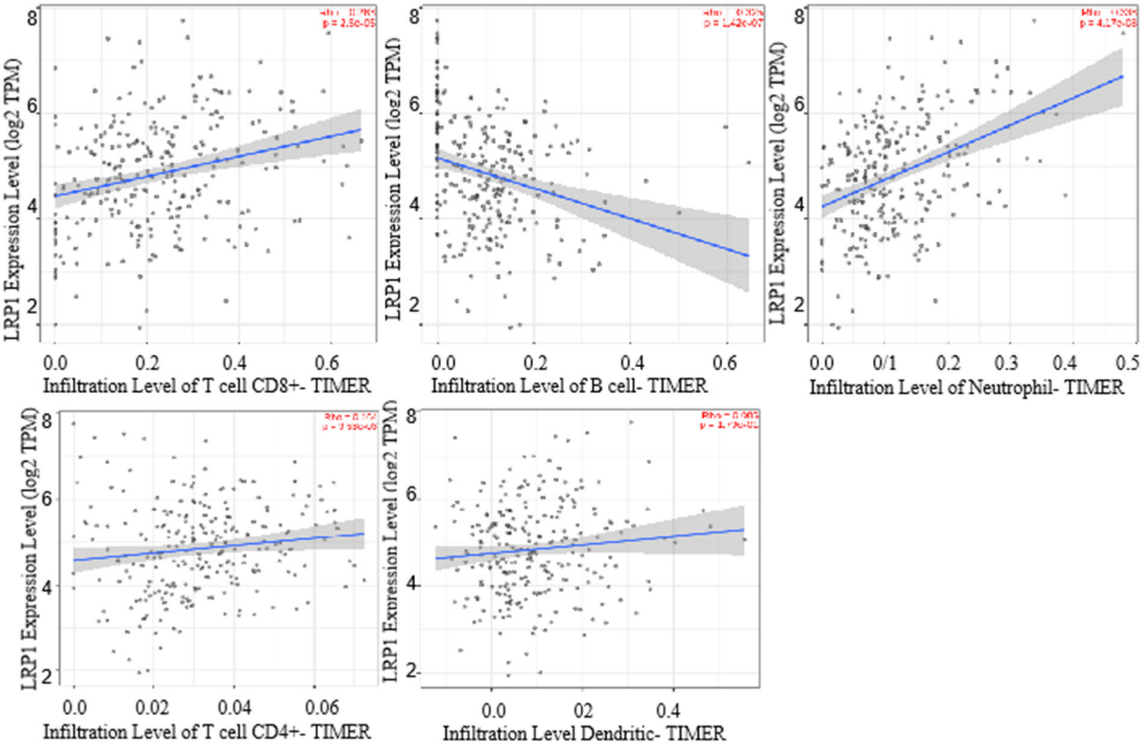

LRP1 Expression Level (log2 TPM)
Infiltration Level of T cell CD8+- TIMER
Infiltration Level of B cell- TIMER
Infiltration Level of Neutrophil- TIMER
Infiltration Level of T cell CD4+- TIMER
Infiltration Level Dendritic- TIMER

B．BLCA

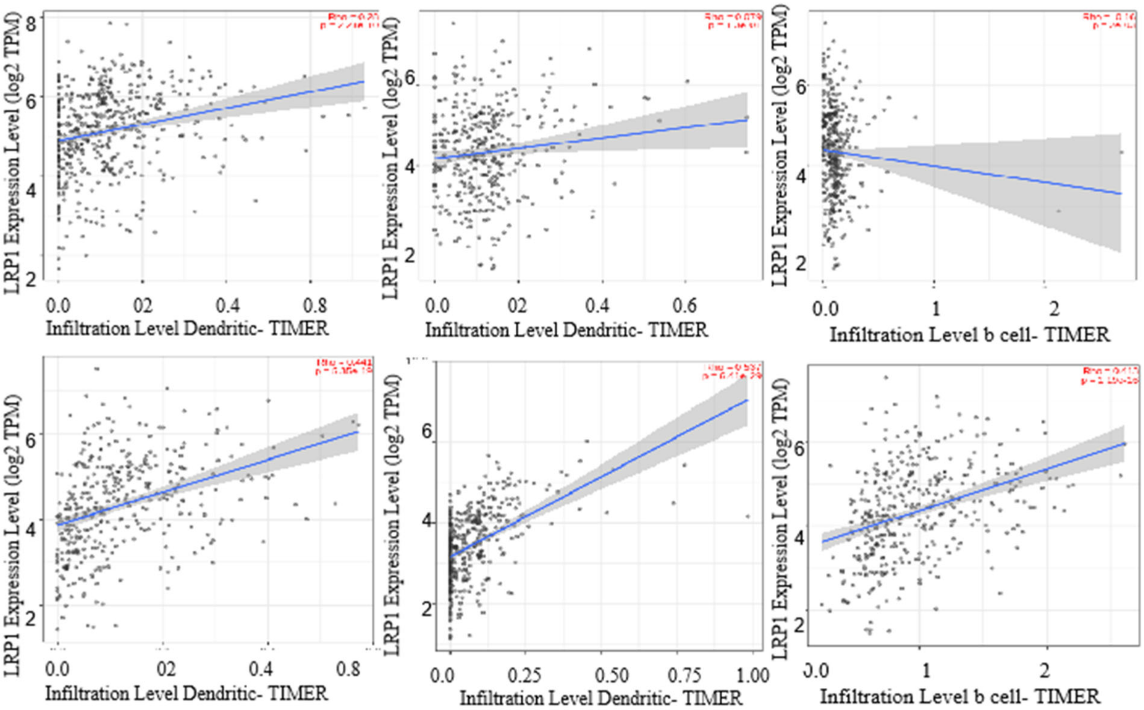

LRP1 Expression Level (log2 TPM)
Infiltration Level Dendritic- TIMER
Infiltration Level Dendritic- TIMER
Infiltration Level b cell- TIMER
Infiltration Level Dendritic- TIMER
Infiltration Level Dendritic- TIMER
Infiltration Level b cell- TIMER

C. GBM

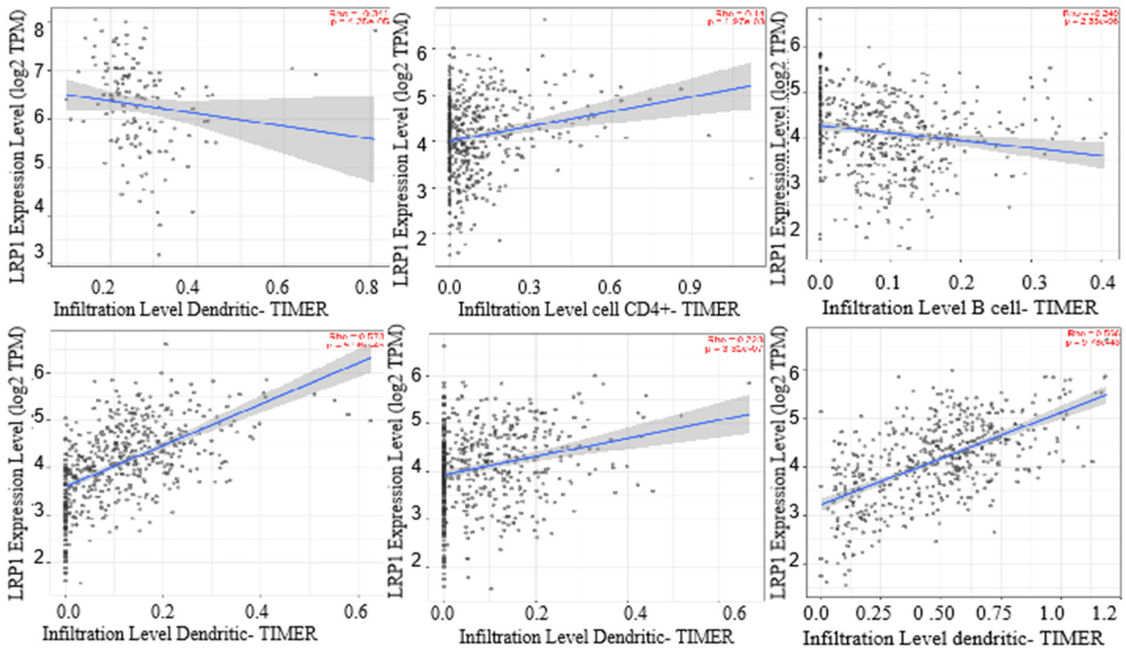
LRP1 Expression Level (log2 TPM)
Infiltration Level Dendritic- TIMER
Infiltration Level cell CD4+- TIMER
Infiltration Level B cell- TIMER
Infiltration Level Dendritic- TIMER
Infiltration Level Dendritic- TIMER
Infiltration Level dendritic- TIMER

D. KIRP

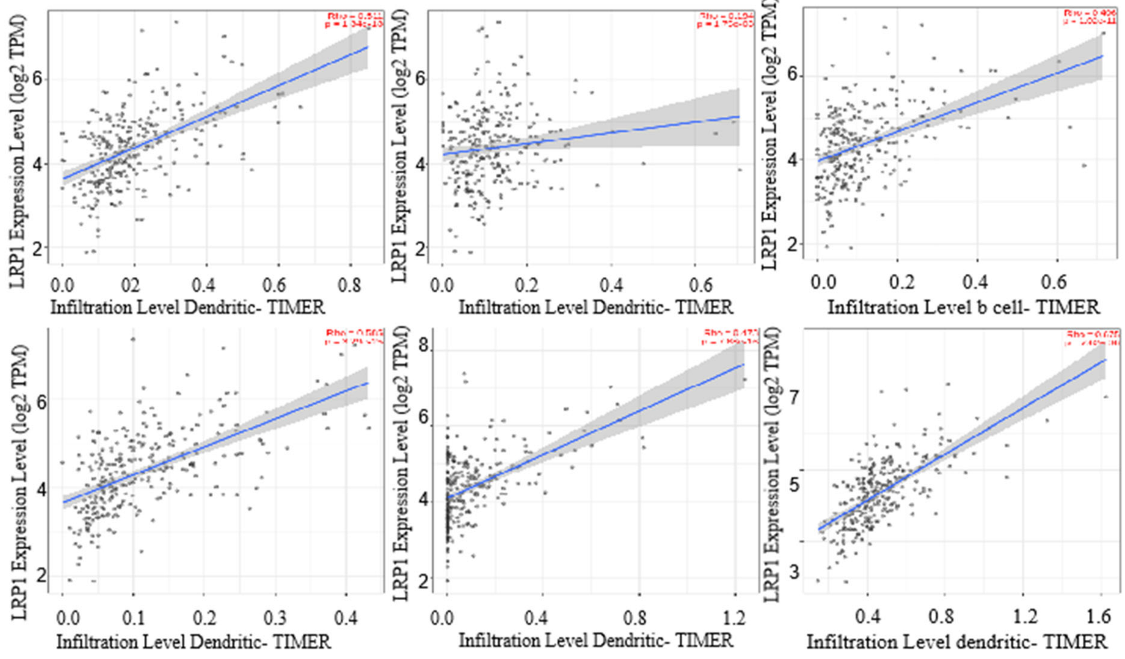
LRP1 Expression Level (log2 TPM)
Infiltration Level Dendritic- TIMER
Infiltration Level Dendritic- TIMER
Infiltration Level b cell- TIMER
Infiltration Level Dendritic- TIMER
Infiltration Level Dendritic- TIMER
Infiltration Level dendritic- TIMER

E. THCA

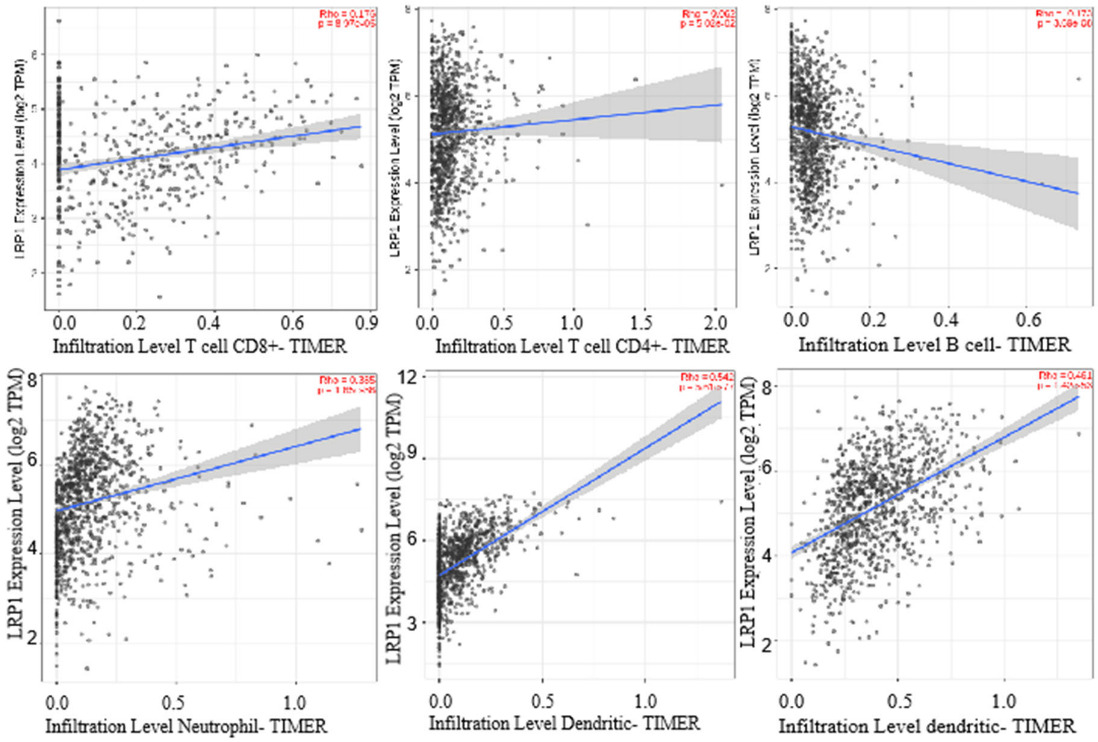
LRP1 Expression Level (log2 TPM)
Infiltration Level T cell CD8+- TIMER
Infiltration Level T cell CD4+- TIMER
Infiltration Level B cell- TIMER
Infiltration Level Neutrophil- TIMER
Infiltration Level Dendritic- TIMER
Infiltration Level dendritic- TIMER

F. KIRC

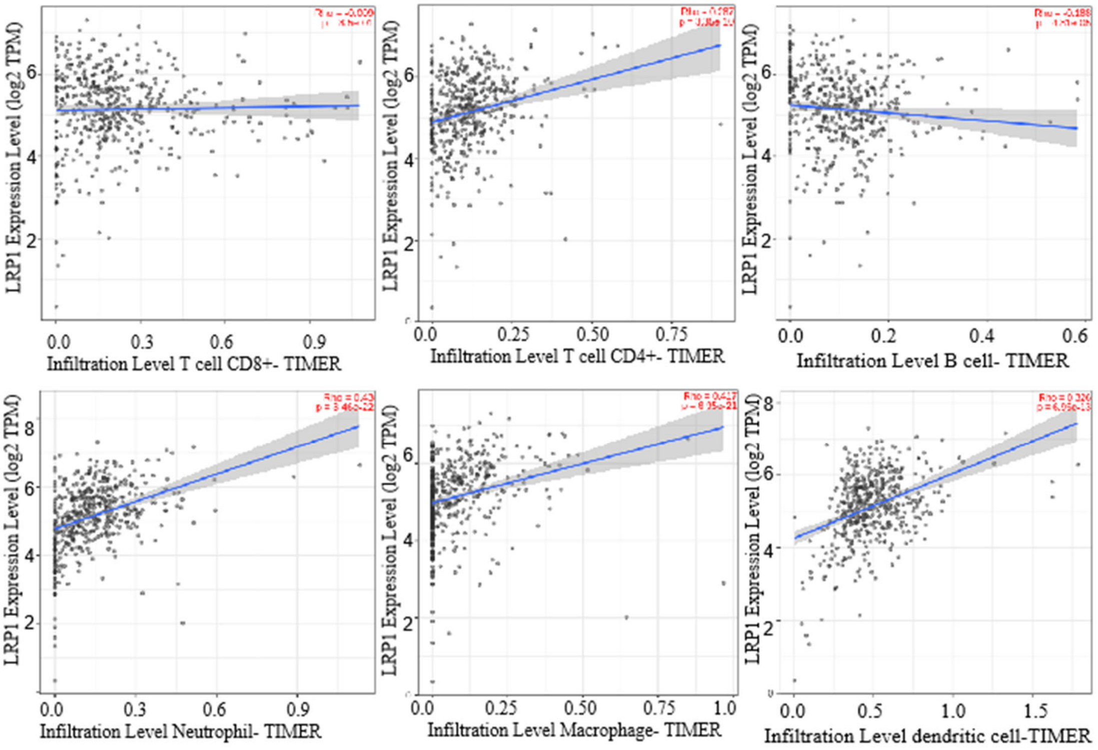
LRP1 Expression Level (log2 TPM)
Infiltration Level T cell CD8+- TIMER
Infiltration Level T cell CD4+- TIMER
Infiltration Level B cell- TIMER
Infiltration Level Neutrophil- TIMER
Infiltration Level Macrophage- TIMER
Infiltration Level dendritic cell-TIMER

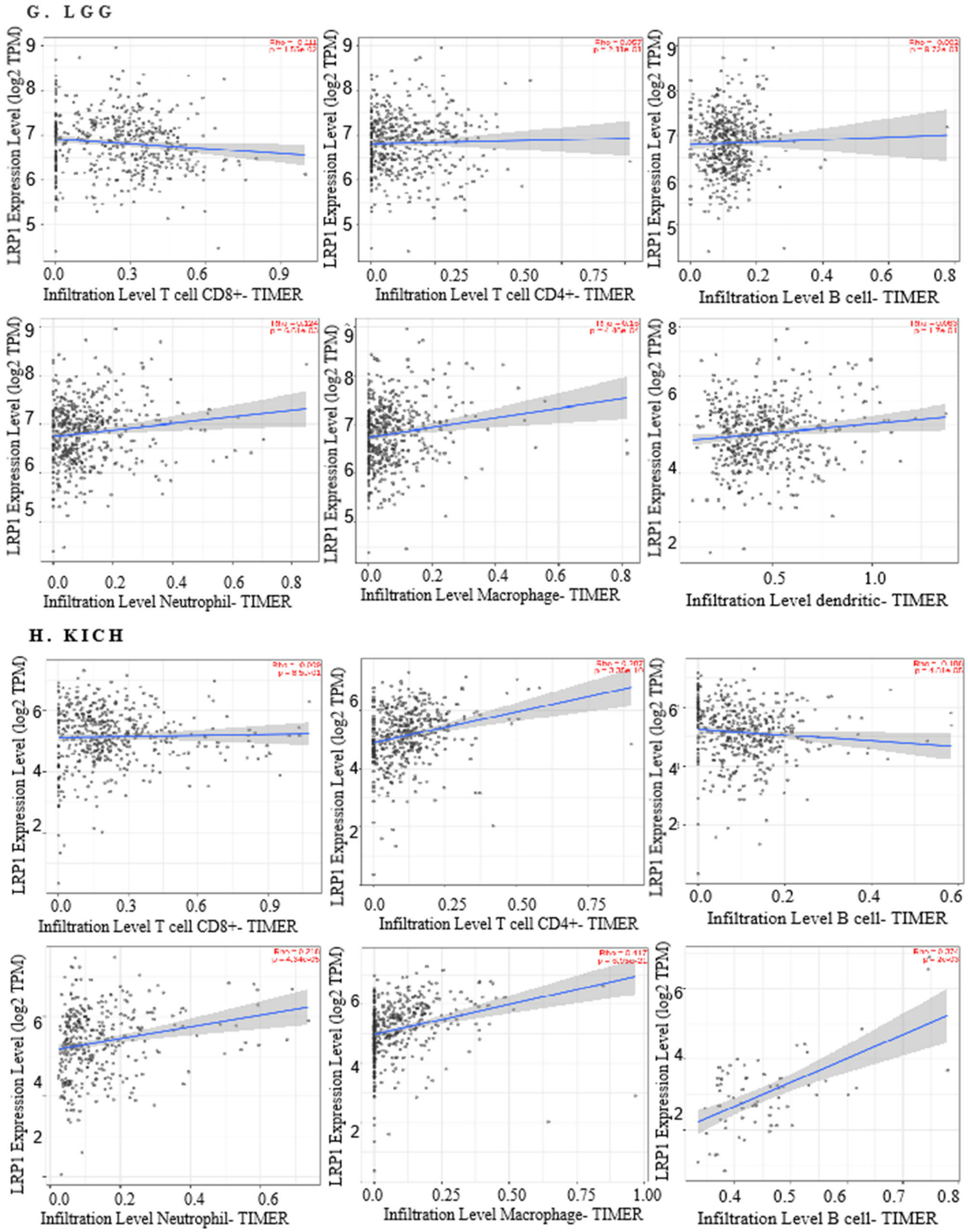

Supplementary Figure S16: The Relationship between LRP1 Expression and Immune Infiltrating Levels in Various Cancer Types: it illustrates the relationship between LRP1 (Low-Density Lipoprotein Receptor-Related Protein 1) expression and immune infiltrating levels in multiple cancer types, including ovarian cancer (OC), bladder cancer (BLCA), glioblastoma (GBM), kidney renal

papillary cell carcinoma (KIRP), thyroid carcinoma (THCA), kidney renal clear cell carcinoma (KIRC), low-grade gliomas (LGG), and kidney renal clear cell carcinoma (KICH).

A. In ovarian cancer, the expression of LRP1 is positively correlated with the tumour infiltrating cells such as CD4+ T cells, CD8+ T cells, neutrophils, dendritic cells, and macrophages, while negatively related to B cells.
B. In bladder cancer, LRP1 expression is positively associated with CD4+ T cells, CD8+ T cells, neutrophils, dendritic cells, and macrophages, and negatively correlated with B cells.
C. In glioblastoma, LRP1 expression shows a positive relationship with CD4+ T cells, neutrophils, dendritic cells, and macrophages, while negatively correlated with CD8+ T cells and B cells.
D. In kidney renal papillary cell carcinoma, LRP1 expression is positively related to CD4+ T cells, CD8+ T cells, neutrophils, dendritic cells, macrophages, and B cells.
E. In thyroid carcinoma, LRP1 expression exhibits a positive correlation with CD4+ T cells, CD8+ T cells, neutrophils, dendritic cells, and macrophages, while negatively associated with B cells.
F. In kidney renal clear cell carcinoma, LRP1 expression is positively related to CD4+ T cells, neutrophils, dendritic cells, and macrophages, while negatively correlated with CD8+ T cells and B cells.
G. In low-grade gliomas, LRP1 expression shows a positive relationship with CD4+ T cells, neutrophils, dendritic cells, and macrophages, while negatively correlated with CD8+ T cells and B cells.
H. In kidney renal clear cell carcinoma, LRP1 expression is positively associated with CD4+ T cells, CD8+ T cells, neutrophils, dendritic cells, and macrophages, while negatively related to B cells.

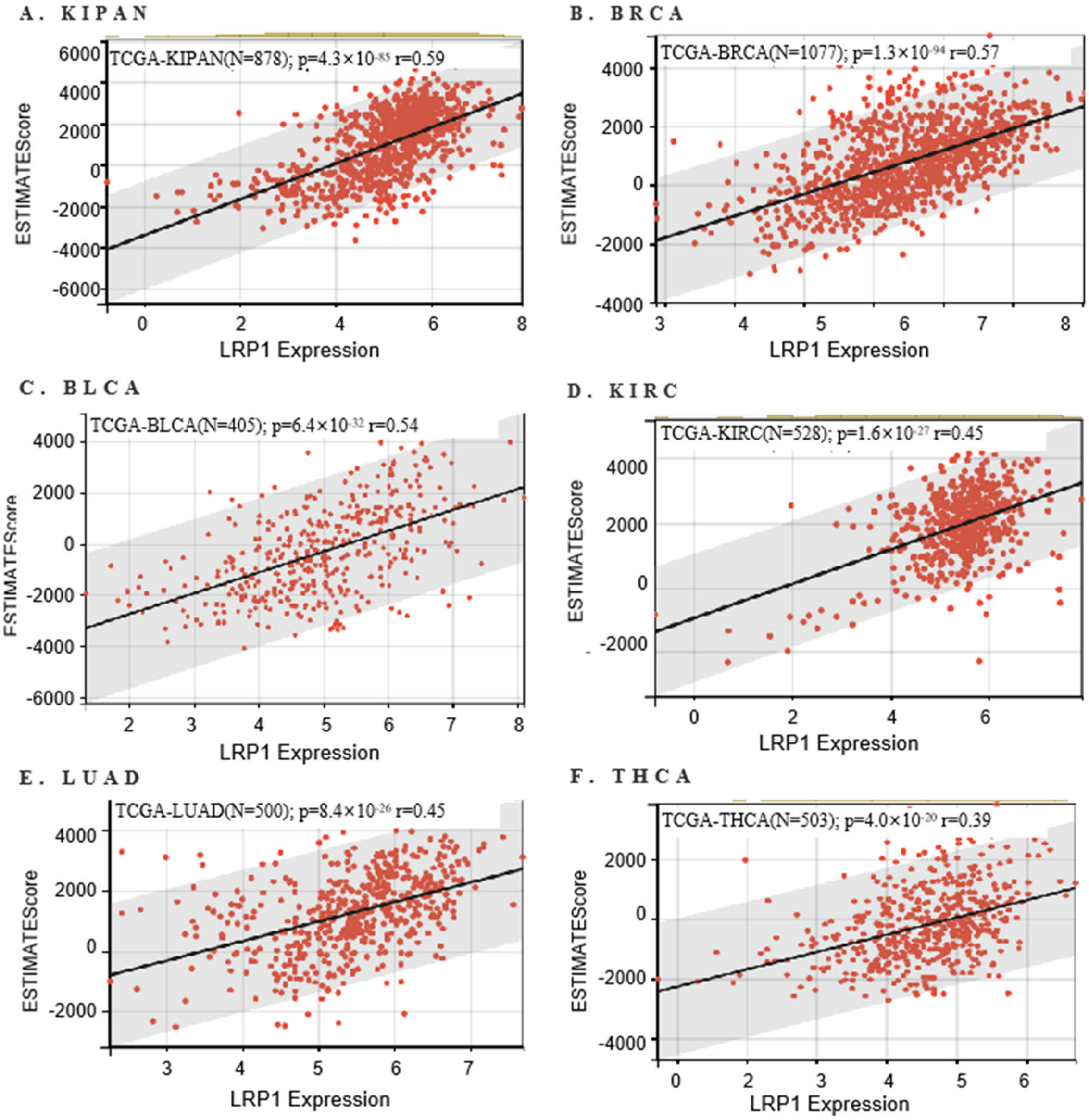
A. KIPAN
TCGA-KIPAN(N=878); p=4.3×10-85 r=0.59
ESTIMATEScore
LRP1 Expression
B. BRCA
TCGA-BRCA(N=1077); p=1.3×10-94 r=0.57
ESTIMATEScore
LRP1 Expression
C. BLCA
TCGA-BLCA(N=405); p=6.4×10-32 r=0.54
ESTIMATEScore
LRP1 Expression
D. KIRC
TCGA-KIRC(N=528); p=1.6×10-27 r=0.45
ESTIMATEScore
LRP1 Expression
E. LUAD
TCGA-LUAD(N=500); p=8.4×10-26 r=0.45
ESTIMATEScore
LRP1 Expression
F. THCA
TCGA-THCA(N=503); p=4.0×10-20 r=0.39
ESTIMATEScore
LRP1 Expression

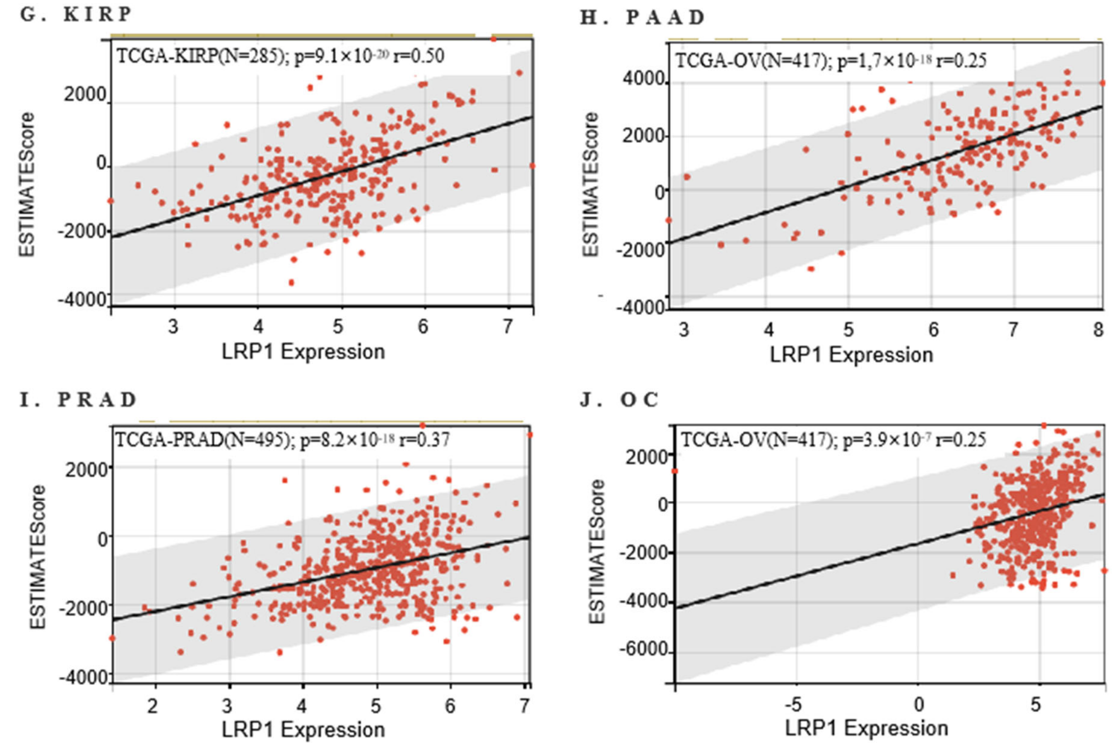

Supplementary Figure S17: Correlation of LRP1 Expression with Estimate Score in Various Cancer Types: displays the correlation between LRP1 expression and Estimate Score (tumour purity) in different cancer types:

A. LRP1 expression was positively related to tumour purity in all cancer types, directly in Kidney Renal Papillary Cell Carcinoma.
B. LRP1 expression was positively correlated with tumour purity in Breast Invasive Carcinoma.
C. LRP1 expression was directly correlated with tumour purity in Bladder Cancer.
D. LRP1 expression was positively correlated with tumour purity in Kidney Renal Clear Cell Carcinoma.
E. LRP1 expression was positively correlated with tumour purity in Lung Adenocarcinoma.
F. LRP1 expression was positively correlated with tumour purity in Thyroid Carcinoma.
G. LRP1 expression was directly correlated with tumour purity in Kidney Renal Papillary Cell Carcinoma.
H. LRP1 expression was directly correlated with tumour purity in Pancreatic Adenocarcinoma.
I. LRP1 expression was directly correlated with tumour purity in Prostate Adenocarcinoma.
J. LRP1 expression was directly correlated with tumour purity in Ovarian Cancer.

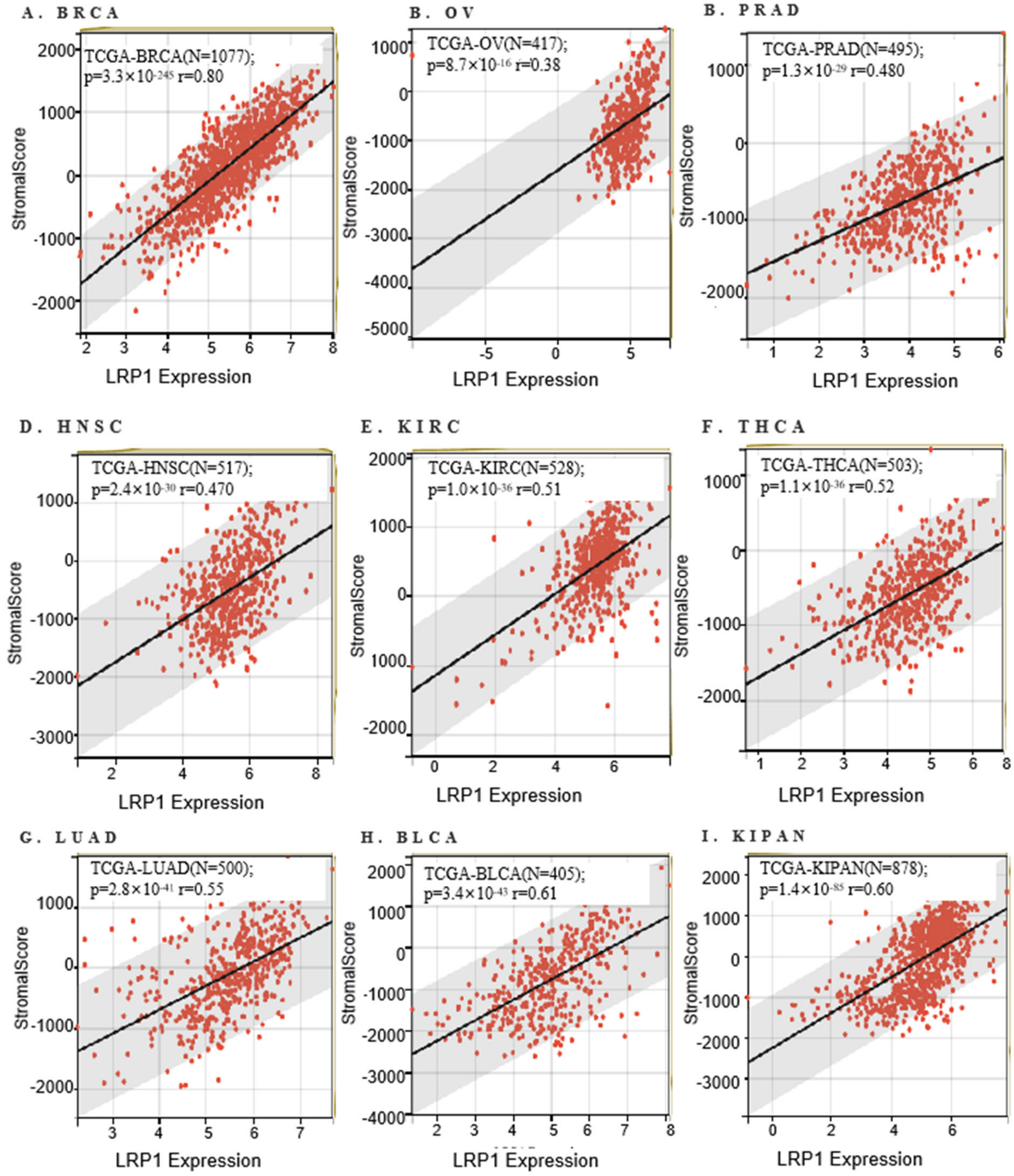

Supplementary Figure S18: Correlation of LRP1 Expression with Stromal Score in Multiple Cancer Types; it illustrates the correlation between LRP1 (Low-Density Lipoprotein Receptor-Related Protein 1) expression and Stromal Score in various cancer types:

A. LRP1 expression positively correlated with tumour purity in Breast Invasive Carcinoma (BRCA).
B. LRP1 expression positively correlated with tumour purity in Ovarian Cancer (OC).
C. LRP1 expression positively correlated with tumour purity in Prostate Adenocarcinoma (PRAD).
D. LRP1 expression positively correlated with tumour purity in Head and Neck Squamous Cell Carcinoma (HNSC).
E. LRP1 expression positively correlated with tumour purity in Kidney Renal Clear Cell Carcinoma (KIRC).

F. LRP1 expression positively correlated with tumour purity in Thyroid Carcinoma (THCA).
G. LRP1 expression positively correlated with tumour purity in Lung Adenocarcinoma (LUAD).
H. LRP1 expression positively correlated with tumour purity in Bladder Cancer (BLCA).
I. LRP1 expression positively correlated with tumour purity in the Pan-Kidney cohort (KIPAN).

Supplementary Table

Supplementary Table S1: Overview of the correlations between 9 IRGs and various cancer-related functional states in OC

IRG	Functional State	Correlation Coefficient	Significance (p-value)
LRP1	DNA Repair	-0.48	≤ 0.001
	Differentiation	0.63	≤ 0.001
	Angiogenesis	0.44	≤ 0.001
	Inflammation	0.36	≤ 0.001
	Stemness	0.33	≤ 0.001
AKT2	DNA Repair	-0.58	≤ 0.001
	Cell Cycle	-0.48	≤ 0.001
	Differentiation	0.58	≤ 0.001
	Angiogenesis	0.54	≤ 0.001
	Inflammation	0.5	≤ 0.001
FGF7	DNA Repair	-0.56	≤ 0.001
	DNA Damage	-0.51	≤ 0.001
	Apoptosis	-0.5	≤ 0.001
	EMT	-0.39	≤ 0.001
	Stemness	0.17	≤ 0.001
FOS	Differentiation	0.25	≤ 0.001
	Stemness	0.23	≤ 0.01
	Inflammation	0.22	≤ 0.01
	Quiescence	0.15	≤ 0.05
IL27RA	Differentiation	-0.64	≤ 0.001
	Angiogenesis	-0.5	≤ 0.001
	Inflammation	-0.47	≤ 0.001
	DNA Repair	0.6	≤ 0.001
	Cell Cycle	0.51	≤ 0.001
OBP2A	Invasion	-0.53	≤ 0.001
	EMT	-0.47	≤ 0.001
	Angiogenesis	0.24	0.001
	Proliferation	0.23	≤ 0.05
	Differentiation	0.22	≤ 0.05
PAEP	DNA Damage	-0.59	0.001
	DNA Repair	-0.56	0.001
	Apoptosis	-0.52	0.001
	Quiescence	-0.46	0.001
	Cell Cycle	-0.42	0.001
PDGFRA	DNA Repair	-0.73	≤ 0.001
	Cell Cycle	-0.47	≤ 0.01
	DNA Damage	-0.46	≤ 0.001
	Angiogenesis	0.45	≤ 0.01
	Differentiation	0.4	≤ 0.01
PI3	Invasion	-0.56	≤ 0.05
	EMT	-0.43	≤ 0.05
	Inflammation	0.41	≤ 0.05
	Apoptosis	0.41	≤ 0.05
	Quiescence	0.33	≤ 0.05

Supplementary Table S2: Abbreviations

Abbreviation	Full name
AUC	Area Under the Curves
CPTAC	Clinical Proteomic Tumor Analysis Consortium
DEG	Deferentially expressed gene
EMT	Epithelial- Mesenchymal Transition
GEO	Gene Expression Omnibus
GEPIA	Gene Expression Profiling Interactive Analysis
GO	Gene Ontology
GDC	Genomic Data Commons
GTE	Genotype Tissue Expression
IC50	Half- maximal inhibitory concentration
K-M	Kaplan- Meier
KEGG	Kyoto Gene and Genome Encyclopedia
OS	Overall Survival
ROC	Receiver Operating Characteristic
TCGA	The Cancer Genome Atlas
TME	Tumor microenvironment
TMB	Tumor Mutation Burden
TILs	Tumor-infiltrating lymphocytes
PRRX1	Paired Related Homeobox 1
PI3	Peptidase inhibitor 3
PDGFRA	Platelet derived growth factor receptor alpha
PD1	Programmed cell death protein 1
PAEP	Progestagen Associated Endometrial Protein
OV	Ovarian Carcinoma
OC	Ovarian cancer
OBP2A	Odorant-binding protein 2A
MEOX2	Mesenchyme Homeobox 2
MAPK	Mitogen-activated protein kinase
LRP1	Low density lipoprotein receptor-related protein 1
IRGs	Immune Related Genes
IL27RA	Interleukin 27 receptor, alpha
IgG	Immunoglobulin G
ICGC	The International Cancer Genome Consortium
FOS	Fos Proto-Oncogene,
FGF7	Fibroblast growth factor 7
ELN	Elastin
CTLA4	Cytotoxic T-lymphocyte-associated protein 4
CRC	Colorectal cancer
CHEA3	ChIP-X Enrichment Analysis 3

Supplementary Table S3: Comparative Analysis of Immune-Related Genes (IRGs) in Prognostic and Diagnostic Value Assessment

IRGs	Prognostic value	univariante cox regression	LASSO Cox risk	Hazard ratio	Diagnostic Index	Oncogenic Activities	B cells	CD4+ T cells	CD8+ T cells	Macrophages	ESTIMATE Score	Stromal scores	Priority out of 12
LRP1	<0.05	0.038	0.001	1.29(1.18-1.41)	AUC: 0.872; p-value: 1.8x 10-8	Differentiation, Stemness, Angiogenesis, Inflammation,	(r= -0.315, p< 0.05)	(r= 0.255, p< 0.001)	(r= 0.631, p< 0.001)	(r= 0.499, p< 0.001)	8.9 × 10-13	1.2 × 10-26-	12
FGF7	<0.05	0.047	0.003	0.76(0.67-0.87)	AUC: 0.688; p-value: 2.5x 10-2	Stemness	(r= -0.163, p< 0.001)		(r= 0.47, p< 0.001)	(r= 0.17, p< 0.01)	7.4 × 10-54	7.4 × 10--97	8
IL27RA	<0.05	0.026	0.002	1.11(1.01-1.22)	AUC: 0.621; p-value: 0.099	Differentiation,Angiogenesis, Inflammation, Stemness							5
PAEP	<0.05	0.029	0.007	0.79(0.68-0.91)	AUC: 0.545; p-value: 0.34						4.0 × 10-5	9.9 × 10--5	5
PI3	<0.05	0.003	0.001	1.09(1.04-1.15)	AUC: 0.604; p-value: 0.27						8.7 × 10-5	0.02	6
OBP2A	<0.05	0.005	0.002	0.86(0.79-0.97)	AUC: 0.644; p-value: 0.085	Angiogenesis,Proliferation, Differentiation;							4
PDGFRA	<0.05	0.03	0.045	0.90(0.84-0.95)	AUC: 0.576; p-value: 0.22	Angiogenesis, Differentiation				(r= 0.354, p< 0.001)	9.9 × 10-24	6.1 × 10--49	7
AKT2	<0.05	0.043	0.006	1.05(0.93-1.19)	AUC: 0.51; p-value: 0.46	Differentiation, Angiogenesis, Inflammation	(r= -0.152, p< 0.05)						6
FOS	<0.05	0.023	0.001	0.92(0.84-0.1.01)	AUC: 0.595; p-value: 0.14	Differentiation Stemness, , Quiescence			(r= 0.266, p< 0.001	(r= 0.209, p< 0.001)	1.1 × 10-5	6.5 × 10--9	8

Footnote: HR: Hazard Ratio; AUC: Area Under the Curve; IRGs: Immune-Related Genes; TME: Tumor Microenvironment; LASSS: Least Absolute Shrinkage and Selection Operator; ESTIMATE: Estimation of Stromal and Immune cells in Malignant Tumor tissues using Expression data; The correlations were evaluated using Spearman's correlation analysis; Significance levels: *p < 0.05, **p < 0.01, ***p < 0.001, ****p < 0.0001

Supplementary Table S4: Summary of the hallmark gene sets: name, Gene ontology, description, number of founder sets and number of genes it contains.

Name	# Genes	Description	Collections	Source organism	Contributor
HALLMARK_COAGULATION	138	Genes encoding components of blood coagulation system; also up-regulated in platelets.	H	Homo sapiens	MSigDB Team
HALLMARK_COMPLEMENT	200	Genes encoding components of the complement system, which is part of the innate immune system.	H	Homo sapiens	MSigDB Team
HALLMARK_EPITHELIAL_MESENCHYMAL_TRANSITION	200	Genes defining epithelial-mesenchymal transition, as in wound healing, fibrosis and metastasis.	H	Homo sapiens	MSigDB Team
GOBP_ACTIN_FILAMENT_BASED_PROCESS	809	Any cellular process that depends upon or alters the actin cytoskeleton, that part of the cytoskeleton comprising actin filaments and their associated proteins.	C5 GO	Homo sapiens	Gene Ontology Consortium
GOBP_AMYLOID_BETA_CLEARANCE	39	The process in which amyloid-beta is removed from extracellular brain regions by mechanisms involving cell surface receptors.	C5 GO	Homo sapiens	Gene Ontology Consortium
GOBP_AMYLOID_BETA_CLEARANCE_BY_CELLULAR_CATABOLIC_PROCESS	8	The process in which amyloid-beta is removed from extracellular brain regions by cell surface receptor-mediated endocytosis, followed by intracellular degradation.	C5 GO	Homo sapiens	Gene Ontology Consortium
GOBP_AMYLOID_BETA_CLEARANCE_BY_TRANSCYTOSIS	7	The process in which amyloid-beta is removed from extracellular brain regions by cell surface receptor-mediated endocytosis, followed by transcytosis across the blood-brain barrier.	C5 GO	Homo sapiens	Gene Ontology Consortium
GOBP_AORTA_DEVELOPMENT	65	Progression of the aorta over time, from its initial formation to mature structure. An aorta is an artery that carries blood from the heart to other parts of the body.	C5 GO	Homo sapiens	Gene Ontology Consortium

GOBP_AORTA_MORPHOGENESIS	38	Process in which the anatomical structures of an aorta are generated and organized. An aorta is an artery that carries blood from the heart to other parts of the body.	C5 GO	Homo sapiens	Gene Ontology Consortium
GOBP_APOPTOTIC_CELL_CLEARANCE	48	The recognition and removal of an apoptotic cell by a neighboring cell or by a phagocyte.	C5 GO	Homo sapiens	Gene Ontology Consortium
GOBP_ARTERY_DEVELOPMENT	111	The progression of the artery over time, from its initial formation to the mature structure. An artery is a blood vessel that carries blood away from the heart to a capillary bed.	C5 GO	Homo sapiens	Gene Ontology Consortium
GOBP_ARTERY_MORPHOGENESIS	81	The process in which the anatomical structures of arterial blood vessels are generated and organized. Arteries are blood vessels that transport blood from the heart to the body and its organs.	C5 GO	Homo sapiens	Gene Ontology Consortium
GOBP_ASTROCYTE_ACTIVATION	25	A change in morphology and behavior of an astrocyte resulting from exposure to a cytokine, chemokine, cellular ligand, or soluble factor.	C5 GO	Homo sapiens	Gene Ontology Consortium

Supplementary Table S5: Clinicopathological Features of Ovarian Cancer Patients.

Clinical Parameters	Variable	Total (TCGA=492 + GEO=96) = 588) female	Percentages (%)
Age	≤60	325	55.30
	>60	263	44.70
Pathological stage	Stage I	2	0.34
	Stage II	37	6.29
	Stage III	471	80.06
	Stage IV	78	13.31
Histological grade	G1	2	0.34
	G2	68	11.56
	G3	499	84.86
	G4	19	2 .16
CA125	≤4	270	45.86
	>4	318	54.14
